# Nanoparticle type-dependent regulation of polydopamine coating formation and wettability

**DOI:** 10.1039/d6ra07246a

**Published:** 2026-10-07

**Authors:** Meysam Saberi, Ebrahim Jalilnia, Rozita Kazemi, Amir Rezvani-Moghaddam, Hossein Yazdani-Ahmadabadi, Mehdi Salami-Kalajahi

**Affiliations:** a Faculty of Polymer Engineering, Sahand University of Technology P.O. Box: 51335-1996 Tabriz Iran arezvani@sut.ac.ir; b Department of Chemical and Petroleum Engineering, University of Calgary 2500 University Dr NW Calgary T2N 1N4 Canada; c Department of Chemistry, University of British Columbia P.O, Box: V6T 1Z3 Vancouver British Columbia Canada

## Abstract

Polydopamine (PDA) has emerged as a versatile surface modification material because of its universal adhesion and ability to tailor surface properties. However, although various nanomaterials have been incorporated into PDA coatings, the influence of nanoparticle morphology on dopamine polymerization, coating formation, and the resulting surface wettability has not been systematically investigated. In this study, four nanoparticles representing three structural dimensionalities, namely carbon quantum dots (CQDs) and silica nanoparticles (SiO_2_) as 0D nanomaterials, halloysite nanotubes (HNTs) as 1D nanomaterials, and graphene oxide (GO) nanosheets as a 2D nanomaterial, were comparatively evaluated as regulators of PDA coating formation. Dopamine oxidation in the solution phase was monitored by UV-vis spectroscopy, while the resulting coatings were characterized using colorimetry as an indicator of film thickness, field-emission scanning electron microscopy (FESEM) to examine the morphology of the films, atomic force microscopy (AFM) to measure surface roughness, and static water contact angle (WCA) measurements to investigate the interaction of water droplets with the surface of the resulting coatings. The results demonstrate that nanoparticle morphology markedly influences dopamine polymerization, PDA deposition, and coating morphology. Among the investigated systems, CQD-PDA coatings exhibited the largest surface aggregates (323 ± 65 nm) and the highest surface roughness (*R*_a_ = 114 ± 5.5 nm, *R*_q_ = 163 ± 19 nm), indicating enhanced coating growth. Subsequent deposition of a thin polydimethylsiloxane (PDMS) layer on the optimized CQD-PDA coating increased the WCA to 119°, while maintaining a WCA of approximately 90° after five ultrasonication cycles and 103° after five water-impact cycles, demonstrating good coating durability. These findings establish nanoparticle morphology as a key parameter governing PDA coating formation and provide a simple, morphology-directed strategy for fabricating functional coatings with controllable wettability.

## Abstract 2

Polydopamine (PDA) has emerged as a versatile surface modification material because of its universal adhesion and ability to tailor surface properties.

## Graphical abstract

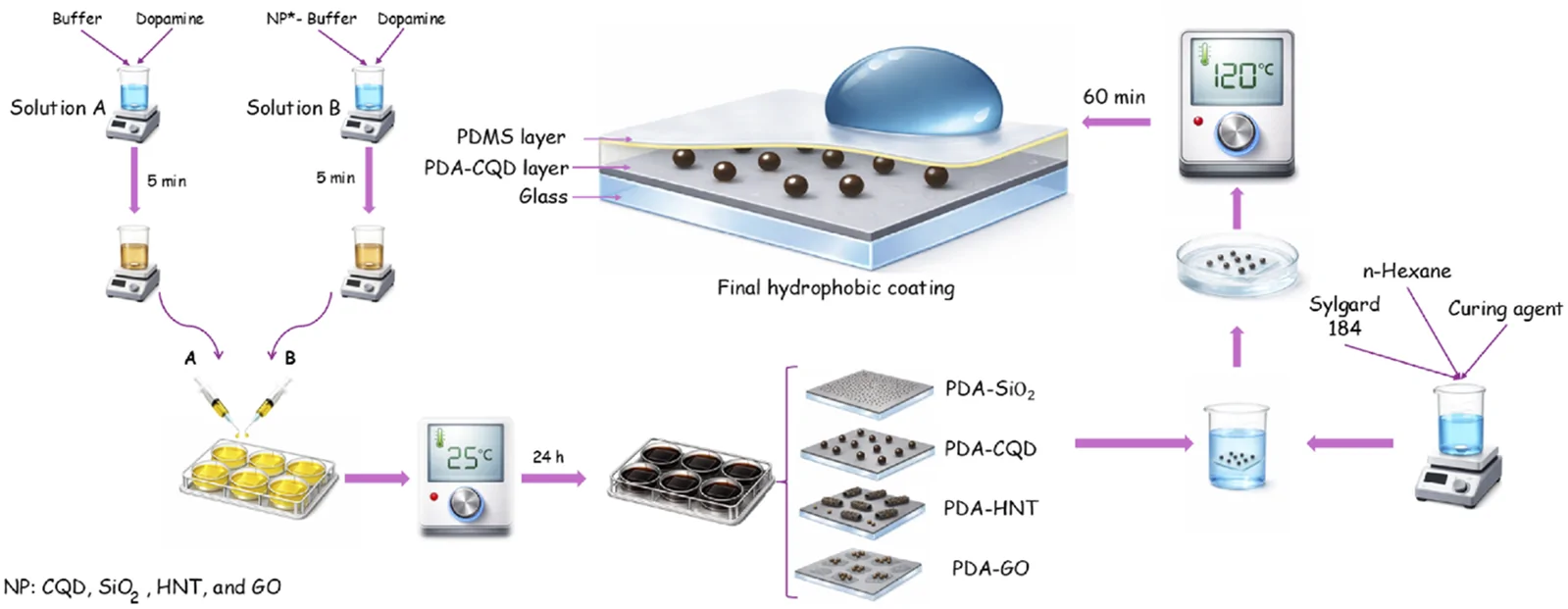

## 1. Introduction

Surface wettability plays a fundamental role in determining the performance of functional coatings by governing the interaction between solid surfaces and liquids. Precise control of wettability is essential for a wide range of applications, including self-cleaning surfaces, corrosion protection, oil–water separation, antifouling materials, and biomedical devices. Depending on the application, surfaces may be designed to exhibit either hydrophilic or hydrophobic characteristics.^1–4^ Hydrophilic surfaces facilitate water spreading and improve interactions with aqueous environments, whereas hydrophobic surfaces provide water repellency, corrosion resistance, and self-cleaning performance. Because wettability is governed by the contrast in surface free energy and surface roughness, engineering these parameters has become a central strategy for developing advanced functional coatings. On rough surfaces, wetting behavior is generally described by the Wenzel and Cassie–Baxter models, in which surface roughness either amplifies the intrinsic wettability or promotes air entrapment beneath the liquid droplet, respectively, resulting in contact angles ranging from superhydrophilic to superhydrophobic states.^5–10^

Because hydrophilic and hydrophobic properties strongly influence the performance and durability of coatings, surface engineering has become a central topic in materials science. In this regard, the choice of suitable surface-modifying materials is crucial. Polydopamine (PDA) has attracted significant attention due to its unique chemical structure and ability to form stable coatings on a wide range of substrates, enabling the tuning of surface properties from superhydrophilic to superhydrophobic and making it attractive for both industrial and biomedical applications.^11,12^

PDA is a bio-inspired polymer generated through the oxidative self-polymerization of dopamine (DA) under mildly alkaline conditions. Owing to the presence of catechol, quinone, amine, and aromatic moieties, PDA has emerged as a highly versatile material for surface modification and functional coatings. These functional groups enable PDA to participate in reactions with nucleophilic species, such as amines and thiols, while simultaneously establishing diverse interactions with a broad range of substrates, resulting in the formation of uniform, stable, and strongly adherent coatings on both organic and inorganic materials.^11,13,14^ A major advantage of PDA is its ability to modify surface chemistry without altering the intrinsic bulk properties of the substrate, distinguishing it from many conventional surface engineering approaches. In addition, PDA exhibits excellent biocompatibility, low cytotoxicity, ionic conductivity, and broadband light absorption. Since its introduction as a universal coating material in 2007, these characteristics have stimulated extensive research and facilitated its application in drug delivery, tissue engineering, anticorrosion systems, antibacterial coatings, and wettability control.^15–17^

PDA can be synthesized by solution oxidation, enzymatic oxidation, or electropolymerization, with solution oxidation being the most widely used because of its simplicity and mild reaction conditions. In this method, dopamine undergoes spontaneous oxidative polymerization in a weakly alkaline solution (typically pH ≈ 8.5) under atmospheric oxygen, producing conformal PDA coatings without the need for harsh conditions or specialized equipment. The outstanding adhesion of PDA originates from a combination of catechol-mediated coordination, hydrogen bonding, covalent coupling, and π-based interactions, while internal crosslinking enhances coating integrity and mechanical stability. Although the exact polymerization mechanism and molecular structure of PDA remain under debate, it is well established that coating formation is highly sensitive to reaction parameters, including pH, buffer composition, oxidants, dopamine concentration, temperature, reaction time, and the presence of additives or nanoparticles. Consequently, these factors govern the morphology, thickness, particle size, and physicochemical properties of PDA coatings, making them highly tunable for advanced surface engineering applications.^13,15,18–20^

As mentioned above, nanoparticles can influence dopamine polymerization and consequently change the structure and performance of PDA coatings. Owing to their high surface-to-volume ratio and unique physicochemical properties, nanoparticles provide abundant active sites that can interact with dopamine and its polymerization intermediates, thereby affecting coating growth and morphology. Depending on their structural dimensionality, nanoparticles are generally classified as zero-dimensional (0D), one-dimensional (1D), and two-dimensional (2D) nanostructures, each offering distinct surface characteristics and interfacial interactions.^21–24^ These differences may significantly influence PDA deposition and the resulting coating properties, highlighting the importance of systematically investigating the role of nanoparticle morphology in PDA-based surface engineering. Because nanoparticle morphology governs surface curvature, specific surface area, and the distribution of accessible reactive sites, it is expected to affect dopamine adsorption, nucleation, and subsequent PDA growth, ultimately influencing the structure and properties of the resulting coatings.

Recent studies have demonstrated that incorporating nanomaterials into PDA coatings is an effective strategy for improving coating performance and broadening their functional applications. Carbon quantum dots have been employed as visible-light-responsive initiators to accelerate dopamine polymerization and produce uniform PDA coatings with enhanced membrane separation performance.^25^ Silica nanoparticles have been combined with PDA to fabricate superhydrophilic and superoleophobic surfaces for efficient oil–water separation, while hybrid systems containing mesoporous silica and graphene oxide have been used to improve the anticorrosion and self-healing performance of epoxy coatings through the adhesive and protective characteristics of PDA.^26,27^ In addition, halloysite nanotubes have provided valuable insights into dopamine polymerization because of their high surface area and negatively charged surfaces, which facilitate the adsorption of reactive intermediates.^28^ More recently, nanoparticle-assisted PDA growth using amine-containing dendrimers has enabled the fabrication of thick and stable PDA coatings, which could be further modified with PDMS to obtain environmentally friendly superhydrophobic surfaces.^29^ PDA-assisted surface modification has also been employed for the fabrication of nanostructured materials containing well-dispersed nonprecious metal nanoparticles. For example, PDA- or polymer-assisted strategies have enabled the incorporation and stabilization of Ni nanoparticles on silica and carbon-based supports, where interactions between metal ions and polymerization processes can promote nanoparticle dispersion and control their size and distribution. Such hybrid structures have been further converted into hollow graphitic carbon spheres and N-doped carbon microtubes with enhanced catalytic performance, particularly for the reduction of 4-nitrophenol.^30,31^ In these systems, PDA can also contribute to the formation of hollow and tubular structures through its interaction with the underlying support, highlighting the important role of substrate characteristics in determining the morphology and distribution of the resulting PDA-based structures.^32^

Collectively, these studies demonstrate the versatility of nanoparticles in tailoring PDA deposition and coating performance. However, most investigations have focused on a single type of nanoparticle for a specific application, making it difficult to distinguish the fundamental role of nanoparticle-morphology-directed polymerization of dopamine, coating formation, and the final film properties. To address this knowledge gap, this study systematically compares four nanoparticles representing three structural dimensionalities, namely carbon quantum dots (CQDs) and silica nanoparticles (SiO_2_) as 0D nanomaterials, halloysite nanotubes (HNTs) as 1D nanomaterials, and graphene oxide (GO) nanosheets as 2D nanomaterials, to elucidate their morphology-dependent effects on dopamine polymerization and PDA coating formation. Furthermore, a thin PDMS top layer was applied to tailor the surface wettability, enabling the fabrication of coatings with tunable wetting behavior ranging from hydrophilic to superhydrophobic. The findings provide new insights into the role of nanoparticle morphology in regulating PDA coating formation and establish a simple and versatile strategy for designing functional surfaces with controllable wettability.

## 2. Experimental

### 2.1 Materials

Dopamine hydrochloride (C_8_H_11_NO_2_·HCl) and tris(hydroxymethyl)aminomethane (Tris) buffer (C_4_H_11_NO_3_) were purchased from Sigma-Aldrich (USA). Citric acid (C_6_H_8_O_7_) and sodium hydroxide (NaOH) were obtained from Merck (Germany). SiO_2_ and HNT (Al_2_Si_2_O_5_(OH)_4_) were also supplied by Sigma-Aldrich (USA). The HNTs were naturally occurring aluminosilicate nanotubes with outer diameter of 30–70 nm, inner diameters of 10–30 nm, and length of approximately 0.5–3 µm. The SiO_2_ nanoparticles were spherical with an average particle size of 10–20 nm and a purity higher than 99%. GO was synthesized in-house at the INKOATLAB laboratory (Iran).^33^ Polydimethylsiloxane (Sylgard 184) with density of 0.965 g cm^−3^ was acquired from Dow Coating (USA). *n*-Hexane (C_6_H_14_) was supplied by Metasan Company from Neutron (Iran), and deionized water was provided by PakAb (Iran).

### 2.2 Hydrothermal synthesis of CQDs

Following procedures similar to previous studies conducted by the INKOATLAB research group,^34^ CQDs were synthesized by dissolving 1 g of citric acid in 50 mL of deionized water at 60 °C under magnetic stirring for 10 min. The homogeneous solution was transferred to a sealed hydrothermal reactor and heated at 160 °C for 16 h. After cooling to room temperature, the product was filtered through a 220 nm polyethylene terephthalate syringe filter and the solid fraction was collected by freeze-drying.

### 2.3 Preparation of buffers

To prepare the Tris buffer solution, Tris buffer powder was dissolved in deionized water under magnetic stirring until a homogeneous solution was obtained. The pH was then adjusted to 8.5 by dropwise addition of sodium hydroxide solution. Finally, distilled water was added to bring the total volume to 500 mL, yielding a 10 mM Tris buffer solution. To prepare buffer containing CQDs, 250 mg of freeze-dried CQDs were added to 40 mL of a pre-prepared buffer solution. The mixture was homogenized using a probe sonicator operating at 80% power for 10 min. The pH was then adjusted to 8.5 by the gradual addition of sodium hydroxide solution. Finally, the buffer solution was added to adjust the total volume to 50 mL, yielding a CQDs concentration of 5 mg mL^−1^. Using the same procedure, buffer solutions containing SiO_2_, HNT, and GO were prepared at the same nanoparticle concentration.

### 2.4 Preparation of pure PDA coatings

PDA coatings were prepared *via* the oxidative self-polymerization of dopamine in alkaline Tris buffer. Dopamine hydrochloride was added to the buffer to a final concentration of 2 mg mL^−1^ and the mixture was stirred at 1000 rpm for 5 min at room temperature to ensure uniform dispersion and to initiate polymerization, as indicated by the solution turning light brown. 3 mL of prepared solution was then dispensed into a 6-well plate containing pre-cleaned glass and incubated at 25 °C for 24 h to allow film growth. After incubation, the solution darkened to brown-black and a dark PDA layer formed on the glass surfaces. The coated substrates were thoroughly rinsed by immersion in deionized water to remove residual, non-adhered species and were stored for subsequent characterization and testing.

### 2.5 Preparation of NP-PDA coatings

To prepare polydopamine coatings incorporating CQDs (PDA-CQD), two solutions with identical dopamine concentrations (2 mg mL^−1^) were prepared. Solution A, serving as the nanoparticle-free reference solution, was prepared by dissolving dopamine hydrochloride in the pre-prepared Tris buffer. Solution B, serving as the nanoparticle-containing solution, was prepared by dissolving the same amount of dopamine hydrochloride in a pre-prepared aqueous CQD-Tris buffer dispersion. Thus, both solutions contained identical concentrations of dopamine hydrochloride and Tris buffer, while only Solution B contained CQDs. Solutions A and B were subsequently mixed at defined volume ratios to obtain a total volume of 3 mL and systematically varied CQD concentrations. This two-solution approach enabled accurate control of the CQD loading while maintaining constant concentrations of the principal dopamine polymerization components. Each resulting mixture was then transferred to a 6-well plate containing pre-cleaned glass substrates for coating deposition. To maintain isothermal conditions during coating formation, the 6-well plate was incubated in an oven at 25 °C for 24 h. Upon completion, the reaction mixture darkened to brown-black, indicating dopamine polymerization, and a dark polydopamine layer formed on the glass substrates. The coated samples were subsequently rinsed by immersion in deionized water to remove residual, unreacted, or weakly adsorbed species and were stored for subsequent characterization and testing.

Using the same approach, polydopamine-based nanocomposite coatings incorporating SiO_2_ (PDA-SiO_2_), HNTs (PDA-HNT), and GO (PDA-GO) were fabricated by mixing nanoparticle-loaded and nanoparticle-free dopamine precursor solutions at predetermined volume ratios, yielding coatings with systematically varied nanoparticle contents. The corresponding mixing ratios for each formulation are summarized in Table 1. A schematic illustration of the preparation process is shown in Fig. 1a.

**Table 1:** Mixing volume ratios of nanoparticle-loaded and nanoparticle-free precursor solutions for each sample

Sample name	PDA-NP^a^ 0.05	PDA-NP 0.1	PDA-NP 0.5	PDA-NP 1	PDA-NP 1.5	PDA-NP 2
Buffer without nanoparticles (mL)	2.97	2.94	2.7	2.4	2.1	1.8
Buffer containing nanoparticles (mL)	0.03	0.06	0.3	0.6	0.9	1.2
Concentration of nanoparticles in sample (mg mL^−1^)	0.05	0.1	0.5	1	1.5	2
DA : NP weight ratio	40 : 1	20 : 1	4 : 1	2 : 1	1.33 : 1	1 : 1

a NP: CQD, SiO_2_, HNT, GO.

**Fig. 1:**
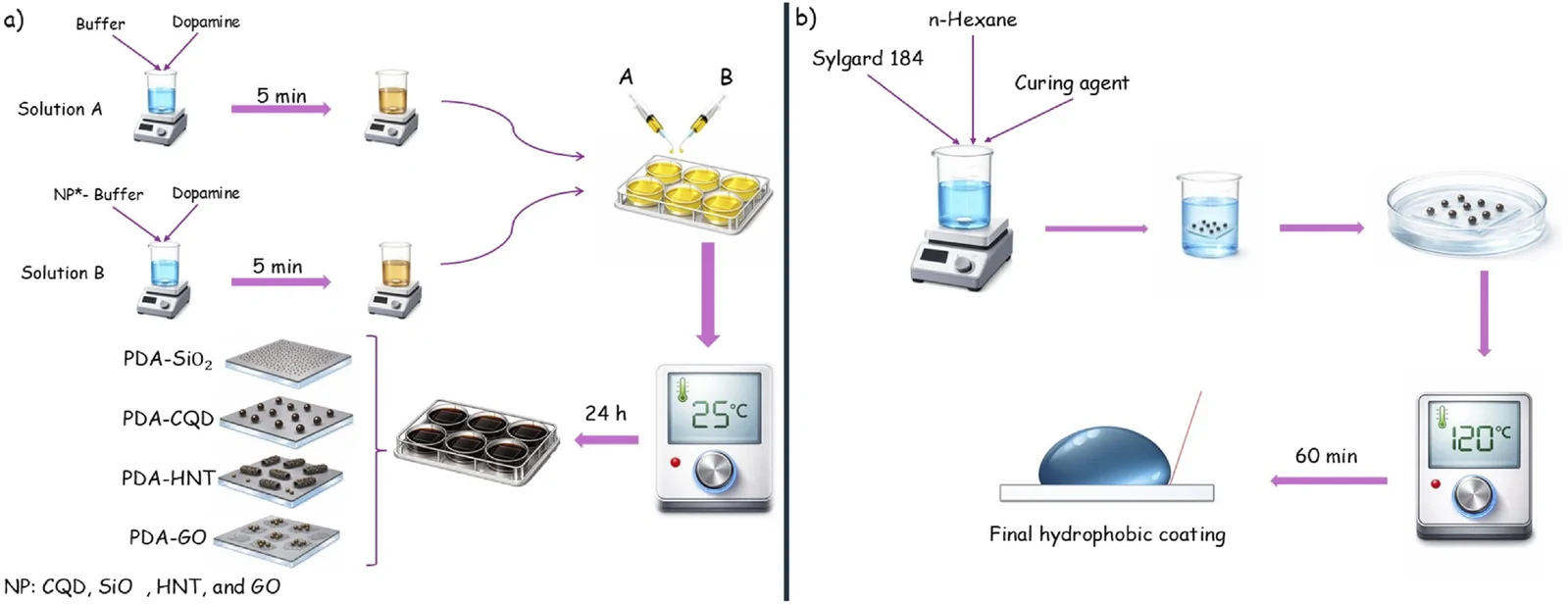
(a) Schematic illustration of PDA-NP coating fabrication and (b) schematic illustration of hydrophobic coatings fabrication.

### 2.6 Preparation of hydrophobic coatings

To obtain hydrophobic coatings, a solution of *n*-hexane, Sylgard 184 and curing agent at a 10 : 1 ratio was first prepared. The optimized samples from the previous steps were immersed in this solution for 5 min, followed by thermal curing in an oven at 120 °C for 60 min. A schematic illustration of the coating procedure is presented in Fig. 1b.

### 2.7 Characterization

Formation of CQDs was verified using a F-2710 spectrofluorometer (Hitachi, Japan); the CQDs were diluted with the corresponding nanoparticle-free buffer to a final concentration of 1 mg mL^−1^, and emission spectra were recorded over 300–700 nm under excitation at 240, 250, 260, and 270 nm. UV-vis measurements were performed to evaluate polymerization in the liquid phase using a i3 spectrophotometer (Hanon, China). The dispersions were diluted 10 : 1 with distilled water and their absorbance was measured in the 380–700 nm range. The wettability of the prepared coatings (hydrophilicity/hydrophobicity) was evaluated by static water contact angle measurements using a CAG-10 (Jikan Company, Iran) goniometer by placing 7 µL water droplets on the surface and determining the angle *via* the circular fitting method. Because the PDA coatings deposited on the glass substrates were extremely thin and translucent, direct thickness determination using conventional thickness measurement techniques was not feasible under the available laboratory conditions. Therefore, the relative coating thickness was evaluated indirectly by colorimetric analysis. CIE L*, a*, and b* color coordinates were measured using an LS176 colorimeter (Linshang, China) equipped with D/8° measurement geometry. Owing to the progressive darkening of PDA during film growth, the CIE L* value was used as a comparative indicator of coating accumulation rather than an absolute measure of film thickness. Since all samples were prepared under identical deposition conditions, differences in L* values were considered to primarily reflect relative variations in PDA deposition. FT-IR spectra were collected on a Invenio-S spectrometer (Bruker, USA) in the 400–4000 cm^−1^ range, using KBr pellets (transmission mode) for powder samples. Surface morphology and particle distribution were examined by FE-SEM Mira3 (TESCAN, Czech Republic). Coated glass specimens were cut and Au-coated to improve surface conductivity, and images were acquired at 5k×, 30k×, and 50k× magnifications. Finally, surface topography was characterized by AFM BRISK (Ara Pazhouhesh, Iran) using a contact-mode probe with an 8 nm tip radius.

## 3. Result and discussion

### 3.1 Characterization of nanoparticles

FTIR spectroscopy was employed to identify the functional groups of the nanoparticles, and the results are presented in Fig. 2. In the spectrum of SiO_2_, a broad band at 3425 cm^−1^ is attributed to the stretching vibration of hydroxyl groups (SiO–H), while characteristic Si–O–Si bands appear in the 1000–1100 cm^−1^ region.^35^ For HNTs, distinct peaks at 3696 and 3618 cm^−1^ correspond to hydroxyl groups located on the inner lumen surface, and the absorption bands at 1000–1100 cm^−1^ are assigned to Si–O–Si linkages.^36^ In the GO spectrum, a broad band at 3400–3500 cm^−1^ is related to hydroxyl groups; peaks at 2924 and 2848 cm^−1^ arise from asymmetric and symmetric stretching of –CH_2_ groups; the peak at 1734 cm^−1^ is associated with carbonyl functionalities; and the band at 1626 cm^−1^ corresponds to C=C stretching.^37^ Overall, these results confirm the high purity of the nanoparticles used in this study. Due to the highly hydrophilic nature of the CQDs, obtaining reliable FTIR measurements for these nanoparticles was not feasible. Since the synthesized CQDs had been previously characterized in detail in INKOATLAB research group,^34^ their characteristic optical properties were used to confirm the successful synthesis and consistency of the prepared nanoparticles. The corresponding optical characterization results are presented in Fig. S1.

**Fig. 2:**
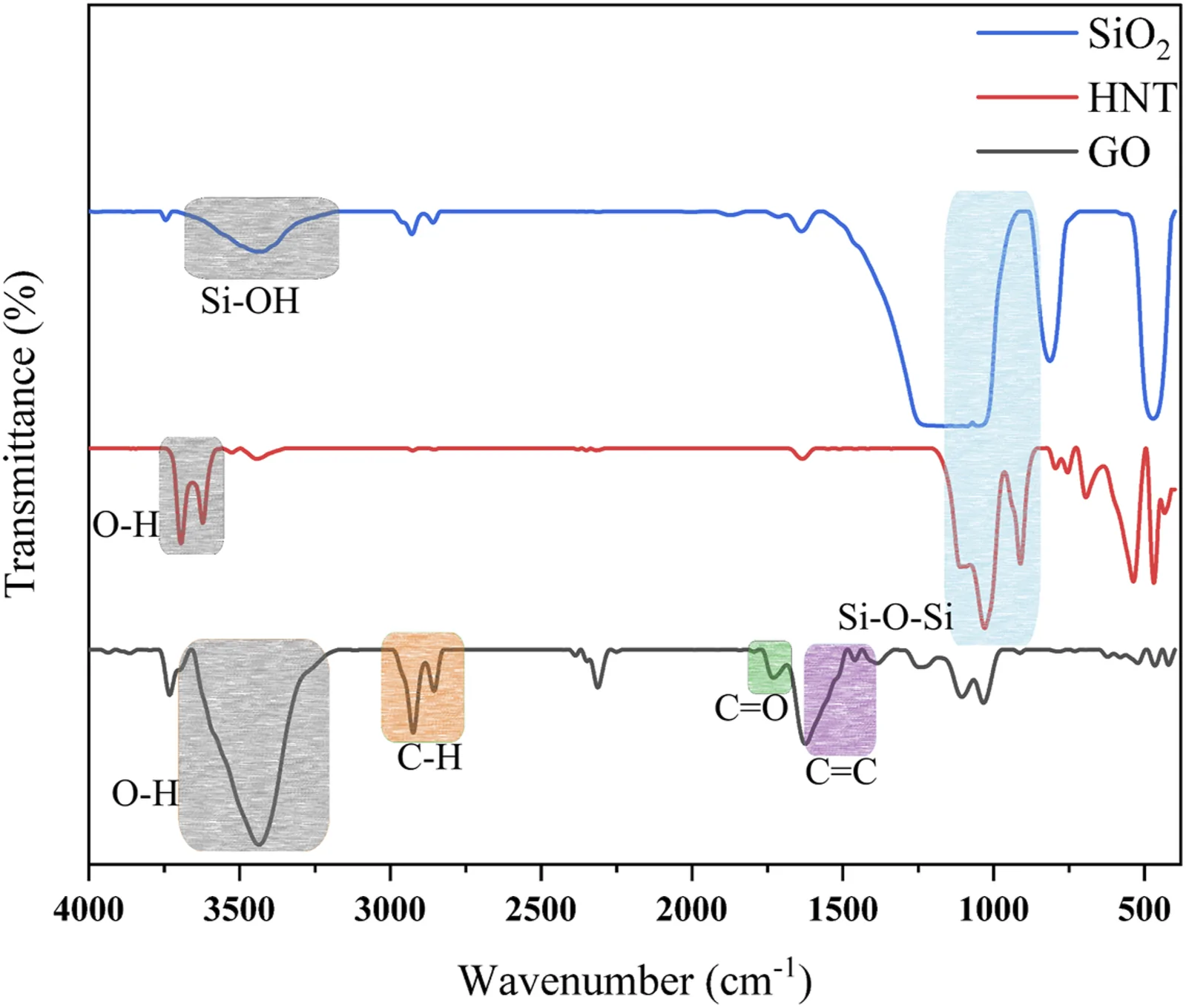
FTIR results of SiO_2_ NP, HNTs, and GO powder.

### 3.2 Studying the PDA polymerization in the liquid phase

The effect of nanoparticles with different morphologies on dopamine polymerization in the liquid phase was investigated using UV-vis spectroscopy. Upon adding dopamine hydrochloride to a buffer solution (pH 8.5), dopamine polymerization was initiated in the presence of dissolved oxygen. This reaction is accompanied by a gradual color change from colorless to dark brown. To quantify this change, UV-vis analysis was performed. In this test, the area under the absorbance–wavelength curve was considered as an index of the sample darkening and, consequently, the extent of polymerization progress.

The UV-vis spectra of PDA-CQD samples prepared with different CQD concentrations are presented in Fig. S2. To account for the intrinsic absorbance of the nanoparticles, the corresponding CQD background was subtracted from each spectrum. All CQD-containing formulations exhibited higher overall absorbance than the nanoparticle-free PDA sample. Notably, the highest CQD loading (2 mg mL^−1^) produced the most pronounced increase in absorbance, indicating a concentration-dependent influence of CQDs on the polymerization process and the optical response of the system. Fig. S3 displays the UV-vis spectra of PDA-SiO_2_ samples after subtraction of the inherent absorbance of SiO_2_ NPs. All PDA-SiO_2_ formulations showed greater absorbance than pure PDA, and a gradual increase in absorbance was observed as the SiO_2_ concentration increased from 0 to 2 mg mL^−1^. These results suggest favorable interactions between SiO_2_ and polydopamine and support their contribution to dopamine polymerization. UV-vis analysis was also carried out for PDA-HNT dispersions containing various concentrations of HNTs (Fig. S4). Following background correction, all samples except the 0.05 mg mL^−1^ formulation exhibited higher absorbance than pure PDA. Moreover, absorbance increased with increasing HNT content, demonstrating the positive role of HNTs in promoting dopamine polymerization in the liquid phase. The UV-vis spectra of PDA-GO samples containing different loadings of graphene oxide nanosheets are shown in Fig. S5, with the intrinsic absorbance of GO subtracted. Polydopamine is expected to deposit onto GO surfaces through noncovalent interactions, including π–π stacking and hydrogen bonding. At GO concentrations below 1.5 mg mL^−1^, no clear change in the absorption profiles was observed, which can be attributed to the limited nanosheet content and insufficient accessible surface area for PDA deposition. In contrast, increasing the GO concentration to 2 mg mL^−1^ resulted in a substantial rise in absorbance, indicating that the higher available surface area facilitates polymerization and enhances polydopamine formation.

Because 5,6-dihydroxyindole (DHI) units and related derivatives are widely regarded as key structural motifs in polydopamine, the absorbance at 420 nm was used as a quantitative indicator of polymerization progress. Fig. 3 compares the absorbance at 420 nm for samples containing different nanoparticle types at concentrations ranging from 0.05 to 2 mg mL^−1^. For all nanoparticles, absorbance increased with increasing concentration, consistent with the spectral trends discussed above. Comparison among nanoparticle types reveals the following order of effectiveness in enhancing dopamine polymerization in the liquid phase: HNT > GO > SiO_2_ > CQD. Overall, the impact of nanoparticles on dopamine polymerization is governed by their morphology, specific surface area, and surface chemistry. HNTs showed the strongest effect, likely due to their tubular/lamellar surface features and diverse functional groups. GO also exerted a significant influence owing to its oxygen-containing functionalities and its capacity for π–π interactions. SiO_2_ exhibited a moderate effect, attributable to surface hydroxyl groups. CQDs, however, produced the weakest enhancement, which may be related to their ultrafine dimensions.

**Fig. 3:**
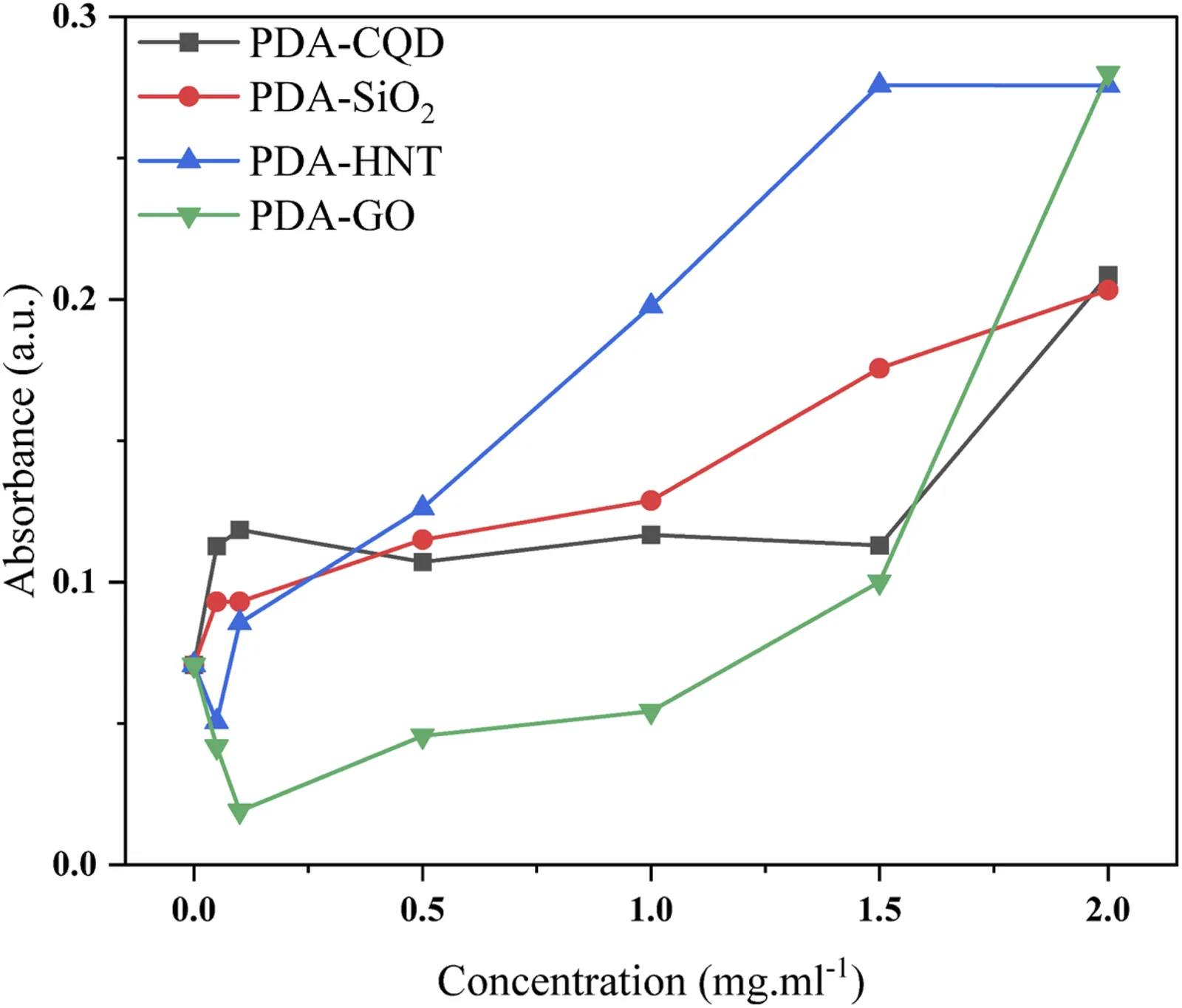
Changes in absorbance at the wavelength of 420 nm for dispersions containing different nanoparticles at concentrations ranging from 0.05 to 2 mg mL^−1^.

For quantitative comparison, the area under the UV-vis absorbance–wavelength curve was calculated for all samples using the trapezoidal rule with a 1 nm step size. The integrated area provides a single quantitative descriptor of the cumulative absorbance across the investigated wavelength range, thereby enabling comparison of the overall spectral absorption response among the samples. The resulting values are summarized in Table 2. For the PDA-CQD formulations, all CQD loadings increased the overall absorbance relative to the nanoparticle-free PDA. The highest CQD concentration (2 mg mL^−1^) produced the largest increase in absorbance intensity, indicating a concentration-dependent contribution of CQDs to the polymerization process and the optical response of the system. For the PDA-SiO_2_ series, the area under curve increased progressively with increasing silica content, reaching its maximum value of 53.74 at 2 mg mL^−1^. This trend clearly demonstrates that the presence of silica nanoparticles and particularly higher loadings enhances dopamine polymerization and increases the amount of polydopamine formed. In line with the common assumption that polymerization occurs preferentially at interfaces and on nanoparticle surfaces, low silica concentrations provide some available area for dopamine adsorption and oxidation; however, the limited number of active sites leads to only modest changes. At higher silica contents, the increased density of surface hydroxyl groups and nucleation sites promotes polydopamine chain growth, resulting in a pronounced increase in optical absorbance. Therefore, silica nanoparticle concentration plays a decisive role in dopamine polymerization under the studied conditions. For PDA-HNT samples, the formulation containing 0.05 mg mL^−1^ HNT exhibited a lower absorbance than pure PDA, whereas all higher concentrations increased absorbance. In particular, increasing the HNT concentration to 1.5 and 2 mg mL^−1^ raised the area under curve to 79 and 77, respectively. This behavior suggests that HNTs, owing to their tubular morphology and high specific surface area, act as effective nucleation platforms for dopamine polymerization. Moreover, surface functional groups on halloysite walls can promote physical and chemical interactions with dopamine, facilitating oxidation and subsequent polydopamine chain propagation. As the nanotube concentration increases, more surface area becomes available, leading to a substantial rise in the amount of formed polydopamine. Hence, HNTs exert a pronounced effect on dopamine polymerization. For GO-containing samples, no substantial change in absorbance was observed over the concentration range of 0.05–1.5 mg mL^−1^. However, the highest absorbance (area under curve = 91) was obtained at 2 mg mL^−1^ GO. These findings indicate that increasing GO loading can markedly enhance absorbance intensity and thus sample darkening, implying a strong concentration-dependent influence of GO on dopamine polymerization in the liquid phase.

**Table 2:** Area under the absorbance–wavelength curve for polydopamine samples containing different nanoparticles at various concentrations

NP concentration (mg mL^−1^)	0	0.05	0.1	0.5	1	1.5	2
Area under curve (a.u.·nm)							
PDA-CQD	20.56	29.58	30.33	25.80	28.58	24.46	54.56
PDA-SiO_2_	20.56	22.51	25.76	31.30	34.43	39.47	53.74
PDA-HNT	20.56	15.77	26.15	38.69	57.03	79.03	77.25
PDA-GO	20.56	10.95	4.65	13.85	15.73	28.01	91.04

### 3.3 Investigation of PDA coatings' thickness

Coating deposition was evaluated indirectly through lightness measurements in the CIE L*a*b* color space. Since PDA becomes progressively darker upon polymerization, a reduction in lightness can serve as a practical proxy for greater optical density and apparent deposition of PDA.^29^ Accordingly, colorimetric analysis was performed and the L*, a*, and b* coordinates were determined. In CIE L*a*b* color space, the L* represents lightness, values approaching 100 correspond to lighter surfaces and, therefore, comparatively lower apparent deposition of PDA. This method enables a quantitative, indirect comparison of apparent deposition among different formulations. The L* values of coatings prepared in the presence of various nanoparticles (0.05–2 mg mL^−1^) are shown in Fig. 4. The CQD-containing coatings exhibited the most pronounced decrease in L*, and the lowest lightness (darkest coating) was obtained for the 1.5 mg mL^−1^ PDA-CQD sample. Although GO and HNTs showed a stronger promoting effect on dopamine polymerization in the liquid phase, they did not yield uniform and mechanically stable coatings on the substrate. This observation is consistent with the accepted dual-pathway mechanism of PDA coating formation: (i) interfacial, *in situ* growth directly on the substrate and (ii) bulk-phase aggregate formation followed by sedimentation and deposition.^38^ In the first pathway, polymerization intermediates anchor to the surface and produce adherent coatings that increase in apparent deposition with time, whereas deposits originating from bulk aggregates typically exhibit weaker adhesion. The present results suggest that nanoparticle addition shifts the process toward bulk aggregation, as dopamine preferentially deposits and polymerizes on nanoparticle surfaces rather than on glass; consequently, a significant fraction of the deposited material is removed during the rinsing step due to weaker interfacial attachment.

**Fig. 4:**
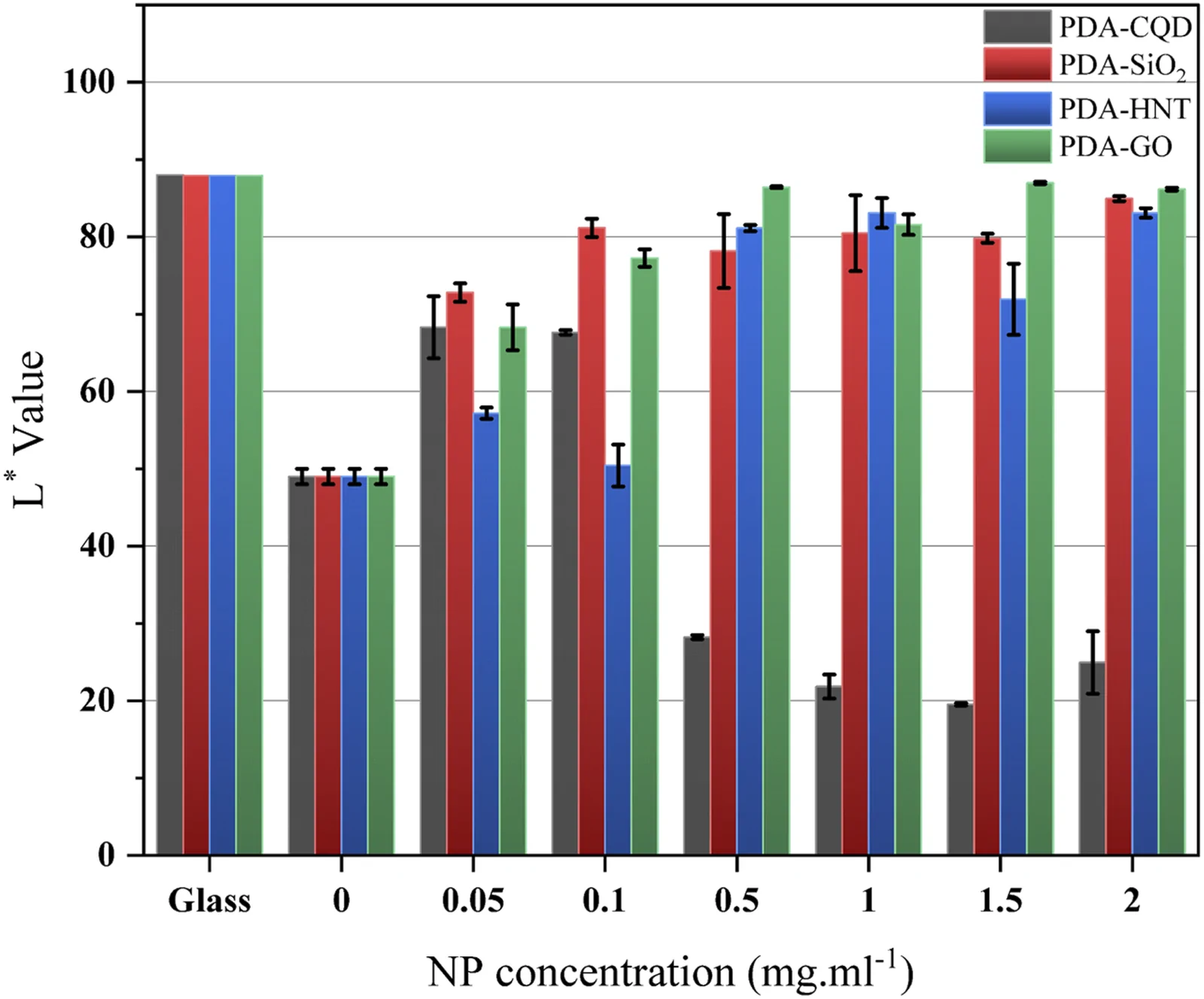
Lightness (L*) of dopamine-coated surfaces in the presence of different nanoparticles at concentrations of 0.05–2 mg mL^−1^.

### 3.4 Studying the wettability of the PDA coatings

To evaluate the wettability of the coatings, static water contact angle (WCA) measurements were performed, and the results are presented in Fig. 5. All nanoparticle-containing coatings exhibited lower contact angles than the pure PDA coating, indicating an overall increase in surface hydrophilicity upon nanoparticle incorporation. Among the investigated systems, the CQD-containing coatings showed the highest hydrophilicity, with the lowest contact angle of 9.3° observed for the 0.5 mg mL^−1^ PDA-CQD sample, corresponding to a superhydrophilic surface. These differences in WCA are consistent with the chemical characteristics identified by FTIR analysis. As shown in Fig. S6, the nanoparticle-containing systems retain the characteristic O–H/N–H stretching band in the 3300–3600 cm^−1^ region, while additional bands associated with the respective nanoparticles are observed. In particular, the CQD-containing system is expected to expose a relatively high density of oxygen-containing surface functionalities, which is consistent with its pronounced hydrophilicity. The ATR-FTIR spectra obtained directly from the coating surfaces (Fig. S7) further confirm the presence of PDA-related functionalities on the outer surface of the coatings, supporting the contribution of surface chemistry to the observed wetting behavior.

**Fig. 5:**
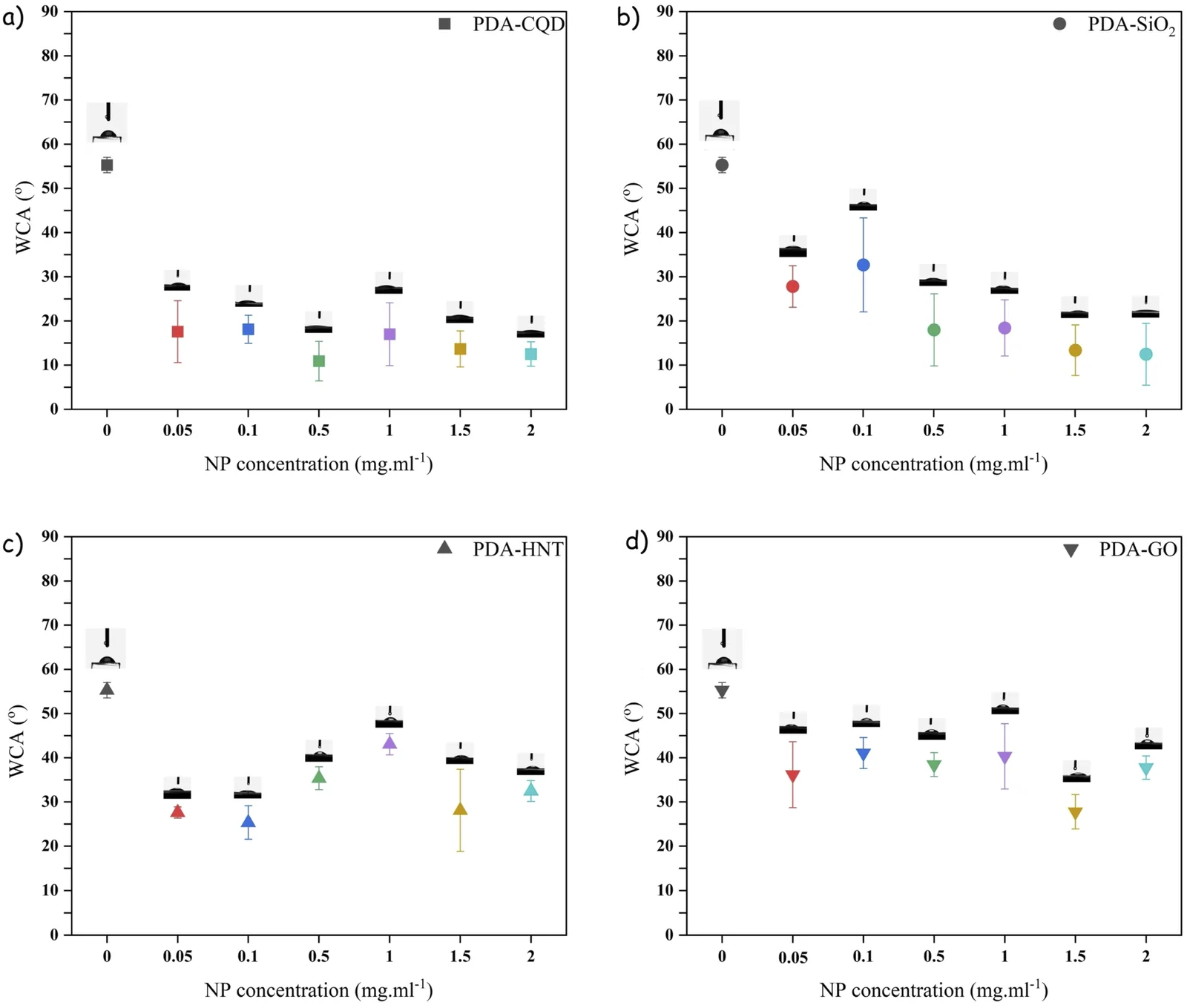
Water contact angle variation as a function of nanoparticle type and concentration (0.05–2 mg mL^−1^) (a) PDA-CQD, (b) PDA-SiO_2_, (c) PDA-HNT and (d) PDA-GO.

The variation in WCA with nanoparticle concentration further indicates that the contribution of surface chemistry is dependent on nanoparticle type. For the CQD- and GO-containing coatings, the contact angle showed relatively limited variation with increasing nanoparticle concentration. This behavior suggests that, once these nanoparticles are incorporated into the outer region of the coating, further increases in loading do not substantially alter the chemical characteristics governing water–surface interactions. This interpretation is supported by the FTIR results, which confirm the presence of the characteristic functional groups of both PDA and the incorporated nanomaterials. For the SiO_2_-containing coatings, in contrast, increasing nanoparticle concentration resulted in a progressive decrease in WCA. This trend is consistent with the increasing contribution of hydroxyl-containing silica surfaces, as evidenced by the characteristic Si–O–Si vibrations observed in the FTIR spectra. Thus, the concentration-dependent increase in hydrophilicity of the PDA-SiO_2_ coatings can reasonably be associated with the increasing contribution of nanoparticle-derived surface functionalities. The PDA-HNT coatings exhibited a non-monotonic variation in WCA with nanoparticle concentration. FTIR analysis confirms the coexistence of PDA-related functionalities and characteristic Si–O–Si vibrations associated with HNTs, indicating that both components contribute to the chemical composition of the coating surface. Therefore, the observed variation in wettability is unlikely to arise from a simple change in the overall chemical composition alone and may also involve changes in the surface organization and distribution of the incorporated HNTs. Such effects can influence the fraction of nanoparticle- and PDA-rich regions exposed to water and, consequently, the apparent contact angle. The morphological evidence presented in the following section provides further insight into these concentration-dependent changes in surface organization.

Overall, the WCA results indicate that nanoparticle incorporation modifies the wettability of PDA coatings, but the magnitude and concentration dependence of this effect are nanoparticle-specific. The FTIR and ATR-FTIR results provide direct evidence that differences in surface chemical composition contribute to these trends, while the subsequent FESEM and AFM analyses are used to further evaluate the role of surface morphology and roughness. Accordingly, the wettability behavior is considered as the combined outcome of surface chemistry and topographical characteristics rather than being attributed exclusively to either factor.

### 3.5 Morphology analysis of PDA coatings

FESEM micrographs of the coatings formed *via* dopamine polymerization in the presence of different nanoparticles (2 mg mL^−1^) at different magnifications are presented in Fig. 6. As observed, the sample containing CQDs (Fig. 6a and e) exhibits semi-spherical particles. This morphology supports the role of CQDs as nucleation sites during the growth of PDA nanoparticles. Consequently, the resulting PDA-CQD nanocomposite also displays a spherical architecture. In addition, a discernible core–shell configuration, which was anticipated based on the proposed mechanism, can be identified in the micrographs. For SiO_2_ samples (Fig. 6b and f), the dominant morphology is likewise spherical. Together with the colorimetric (CIE L*a*b*) results, these observations suggest that PDA growth and deposition occur primarily on the SiO_2_ surfaces rather than glass. However, the reduced coating uniformity at larger length scales indicates weak interparticle cohesion among the formed nanocomposites and limited adhesion to the PDA layer attached to the glass substrate. As a result, a portion of the deposited particles is removed during rinsing, leading to a thinner and less dark coating. In the PDA-HNT system (Fig. 6c and g), FESEM images reveal that PDA nanoparticles preferentially accumulate and grow on the HNT surfaces rather than directly on the glass substrate. Pronounced secondary aggregates of PDA deposited on HNTs are also evident. In contrast, the GO-containing samples indicate comparatively weaker interactions between PDA nanoparticles and GO relative to the other nanoparticles, such that PDA largely forms aggregates in the liquid phase before settling onto GO sheets. Nevertheless, FESEM observations suggest that PDA exhibits a higher affinity for GO than for glass; therefore, coatings formed in the presence of GO tend to be thinner and, consistent with the L*a*b* measurements, exhibit higher lightness than pure PDA coatings.

**Fig. 6:**
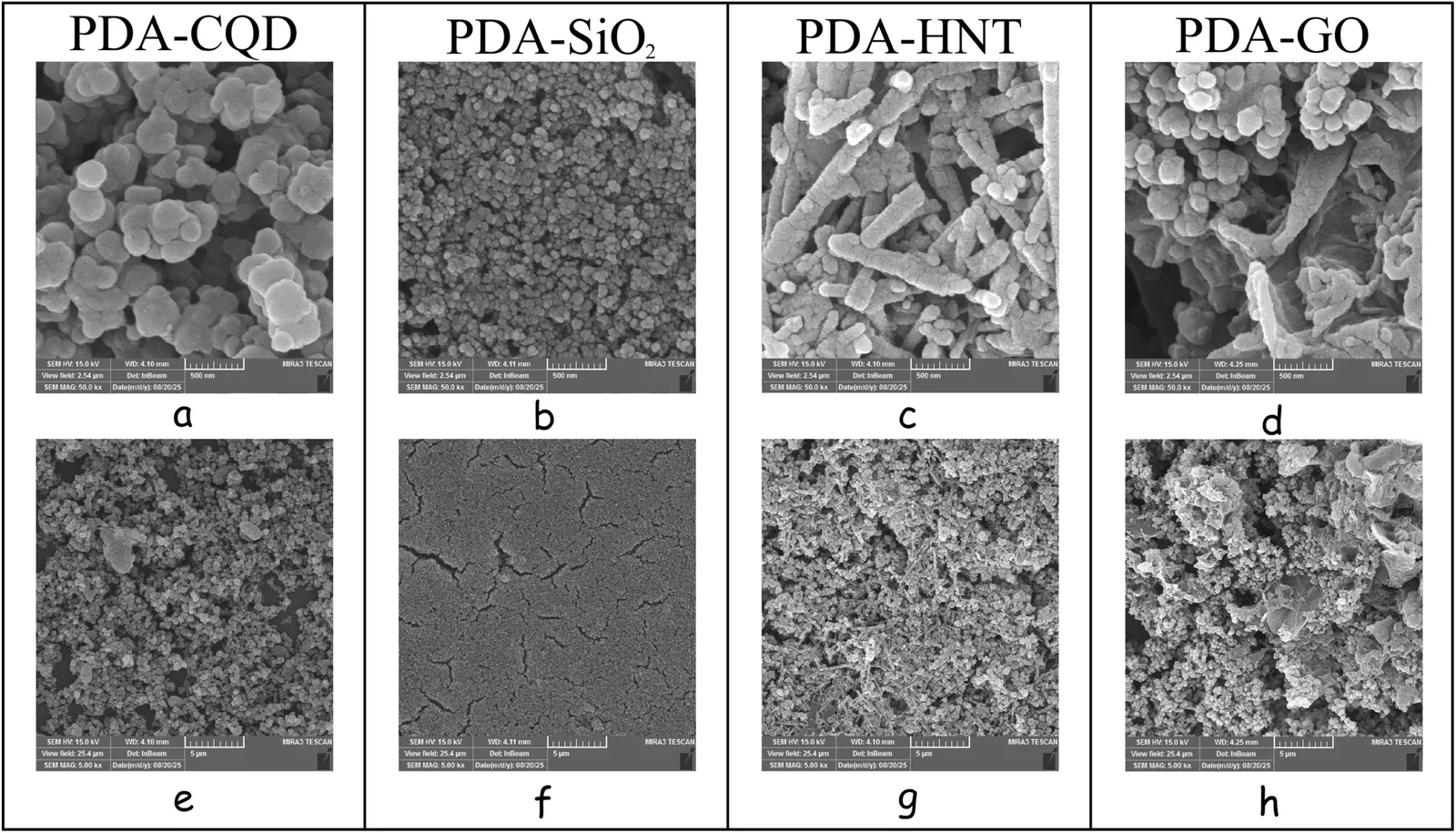
FESEM micrographs of the coatings prepared with polydopamine in the presence of nanoparticles: (a) and (e) CQD, (b) and (f) SiO_2_, (c) and (g) HNT, and (d) and (h) GO.

In addition, the particle size of polydopamine formed in the presence of nanoparticles was quantified by image analysis using ImageJ software, and the results are summarized in Table 3. According to these data, the largest particles were observed for the PDA-CQD sample, with an average particle size of 323 ± 65 nm.

**Table 3:** Particle size of PDA in presence of different nanoparticles

Sample	PDA-CQD	PDA-SiO_2_	PDA-HNT		PDA-GO
			Length	Diameter	
Particle size (nm)	323 ± 65	72 ± 11	618 ± 190	151 ± 26	181 ± 50

Considering the overall FESEM observations and integrating them with the preceding results, particularly the water contact angle measurements and the optical darkening (lightness) analysis, it can be concluded that the PDA coatings prepared in the presence of CQDs and SiO_2_ at 0.5 and 2 mg mL^−1^ exhibit more favorable characteristics than the other formulations. Specifically, these samples showed comparatively thicker and more homogeneous coatings, indicating improved coating formation and stability. Accordingly, they were selected as the optimal candidates for subsequent investigations, including surface roughness analysis and the fabrication of hydrophobic coatings.

### 3.6 Surface roughness analysis

To assess the surface roughness of the prepared coatings, AFM was performed, and the corresponding topography maps are presented in Fig. 7. In addition, roughness parameters were statistically extracted using Gwyddion software, and the results are reported in Table 4. Based on the AFM analysis, the final PDA-SiO_2_ coatings cannot be considered significantly rough. This behavior is consistent with the FESEM observations, which indicate the formation of relatively small particle size and a comparatively smooth coating morphology. In contrast, the CQD-containing coatings exhibited a marked increase in surface roughness. This enhancement can be attributed to stronger interfacial interactions and improved adhesion of the PDA-CQD structures to the substrate. Moreover, FESEM images revealed larger particle sizes and a more pronounced particulate texture in these coatings, which likely contributes directly to the increased roughness measured by AFM.

**Fig. 7:**
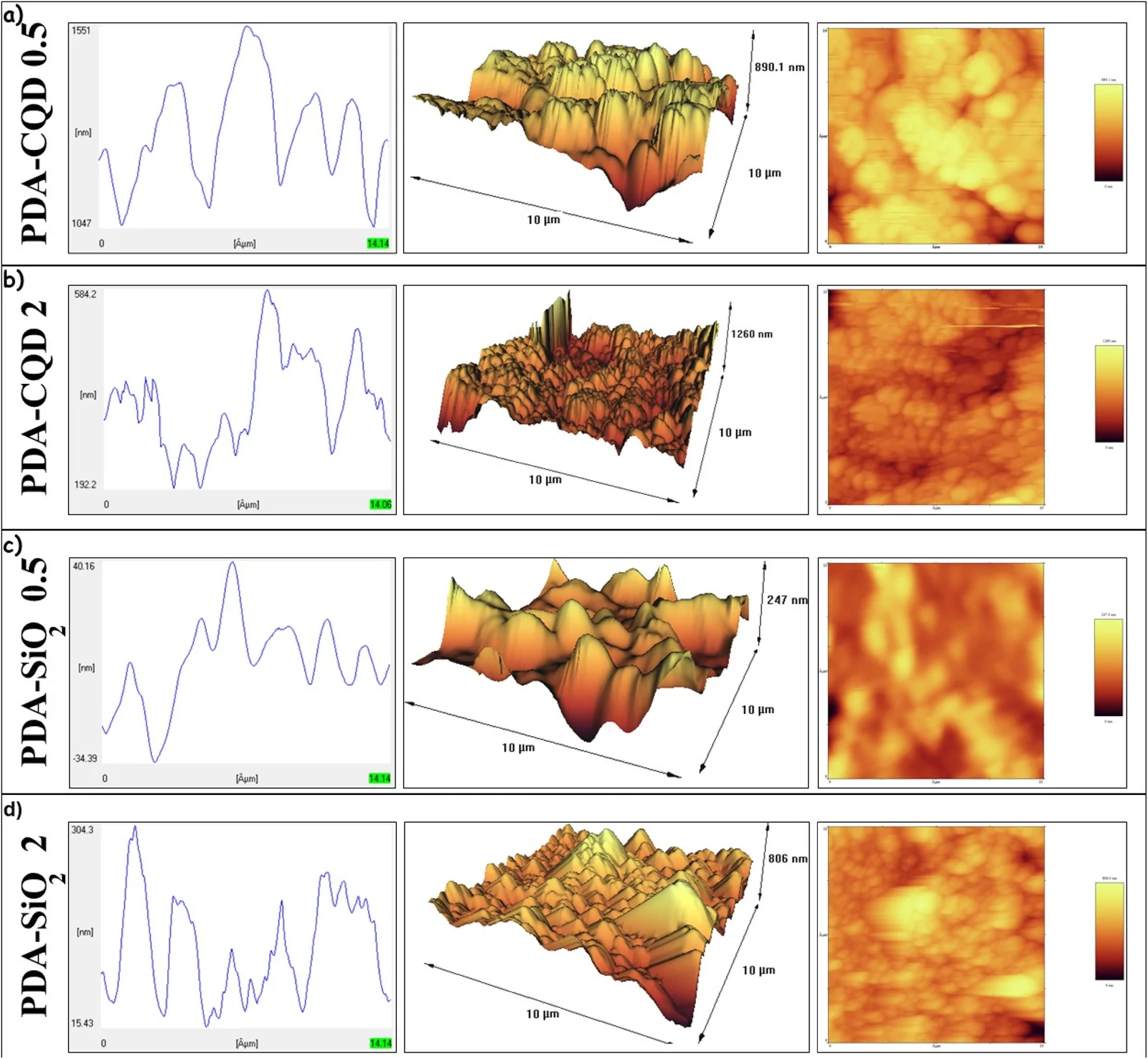
AFM results of samples (a) PDA-CQD 0.5, (b) PDA-CQD 2, (c) PDA-SiO_2_ 0.5, and (d) PDA-SiO_2_ 2.

**Table 4:** Surface roughness parameters of samples

Sample	PDA-CQD 0.5	PDA-CQD 2	PDA-SiO_2_ 0.5	PDA-SiO_2_ 2
*R*_a_ (nm)	90 ± 6.5	114 ± 5.5	63 ± 10.0	67 ± 14.8
*R*_q_ (nm)	126 ± 9.7	163 ± 19.0	84 ± 12.3	92 ± 21.4

Given the Wenzel and Cassie–Baxter wetting models, which predict that increased surface roughness can enhance apparent water repellency, the PDA-CQD sample with 2 mg mL^−1^ of CQDs was selected as the most suitable candidate for the fabrication of hydrophobic coatings.

### 3.7 Formation of hydrophobic coatings

Final hydrophobic coatings were fabricated by applying a PDMS top layer onto the optimized substrate (PDA-CQD2). To benchmark the coating performance, control samples were also prepared, including bare glass and pure PDA-coated glass without nanoparticles. The water repellency of all samples was evaluated by static water contact angle measurements, and the results are presented in Fig. 8. According to the contact angle data, the PDMS-coated bare glass exhibited a water contact angle of 88.6°, while the PDMS-coated PDA coating (without nanoparticles) showed a slightly higher value of 90.1°. In contrast, the sample prepared using the optimized PDA-CQD2 layer followed by PDMS displayed a contact angle of 119°, which falls within the hydrophobic regime. The substantial increase in contact angle can be attributed to the synergistic contribution of two factors: (i) enhanced surface roughness induced by the CQD-assisted PDA micro/nanostructure, as discussed in the previous section, and (ii) reduced surface energy provided by the PDMS top layer. Collectively, these effects shift the surface wetting behavior toward hydrophobicity in the final coating.

**Fig. 8:**
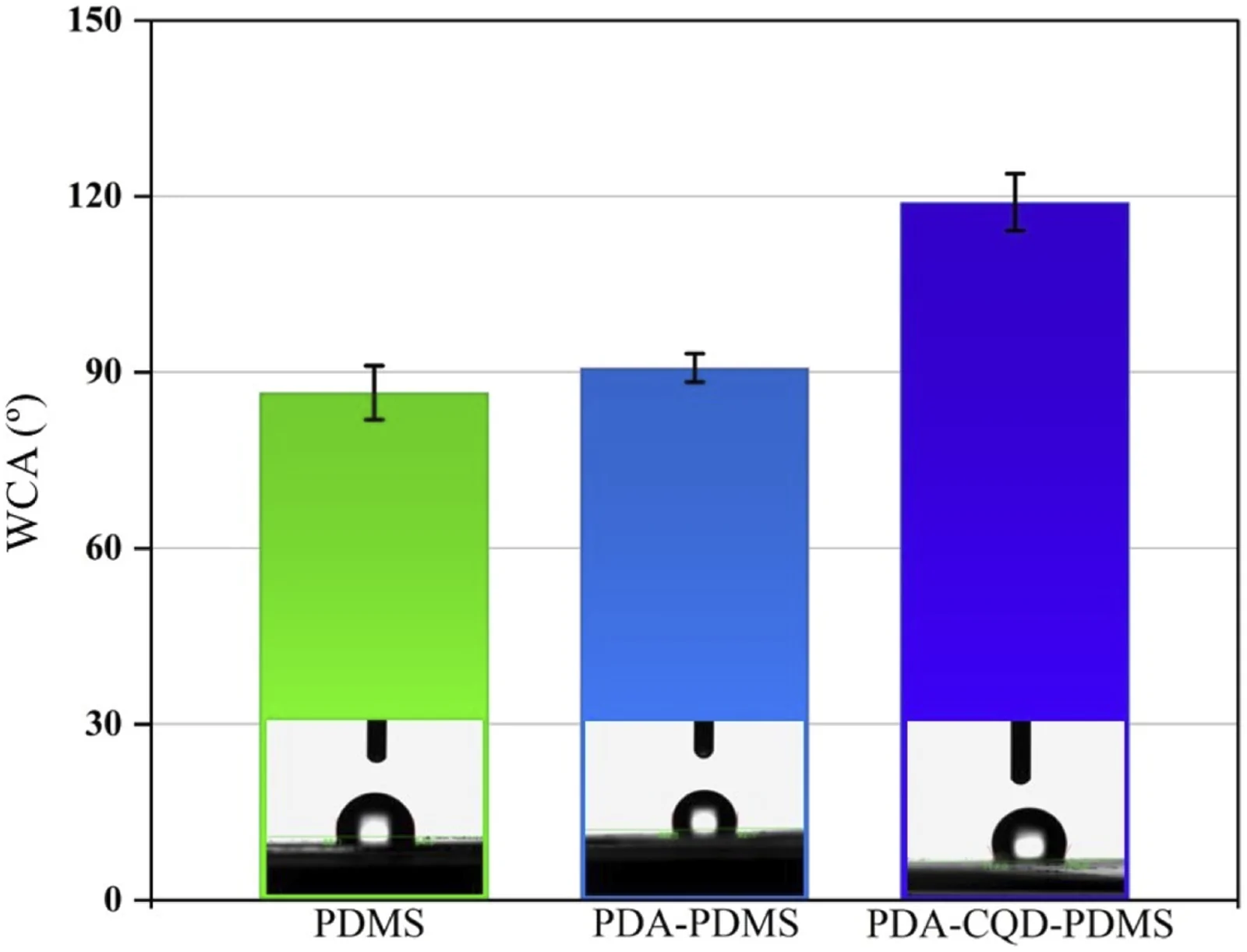
WCA for glass coated with PDMS, PDA-PDMS, and PDA-CQD-PDMS.

### 3.8 Coatings' stability analysis

In the final step, the durability and stability of the prepared coatings were evaluated using accelerated ultrasonic-bath and water-impact tests. In the first test, the samples were immersed in an ultrasonic bath in 1 min cycles, and after each cycle the water contact angle was measured as an indicator of changes in surface wettability. In the second test, designed to simulate repeated droplet impingement, the samples were positioned at a 45° inclination and water was released from a height of 50 cm onto the surface. In each cycle, 25 mL of water was poured onto the sample, and the contact angle was recorded at the end of every cycle. These measurements were used as quantitative metrics to assess coating stability under mechanical loading and environmental exposure.

The evolution of water contact angle after five 1 min ultrasonication cycles is shown in Fig. 9a. As can be seen, the contact angle decreased from the initial value of 119° to approximately 90° after the first cycle and then remained relatively constant around this value up to the fifth cycle. This behavior indicates that ultrasonication caused a pronounced initial reduction in hydrophobicity, followed by a relatively stable contact angle during subsequent cycles. However, the decrease to approximately 90° suggests that part of the surface features contributing to the initial hydrophobicity may have been affected by the first ultrasonication cycle. The contact angle results after five cycles of the water-impact test are presented in Fig. 9b. The coatings retained their hydrophobic character after repeated water droplet impacts, although a gradual reduction in contact angle was observed. After the fifth cycle, the contact angle decreased to approximately 103°, which still corresponds to a hydrophobic surface. These results indicate that the coating maintained a substantial portion of its initial hydrophobicity under the applied intermittent mechanical loading, while also demonstrating some loss of surface hydrophobicity after repeated water impacts.

**Fig. 9:**
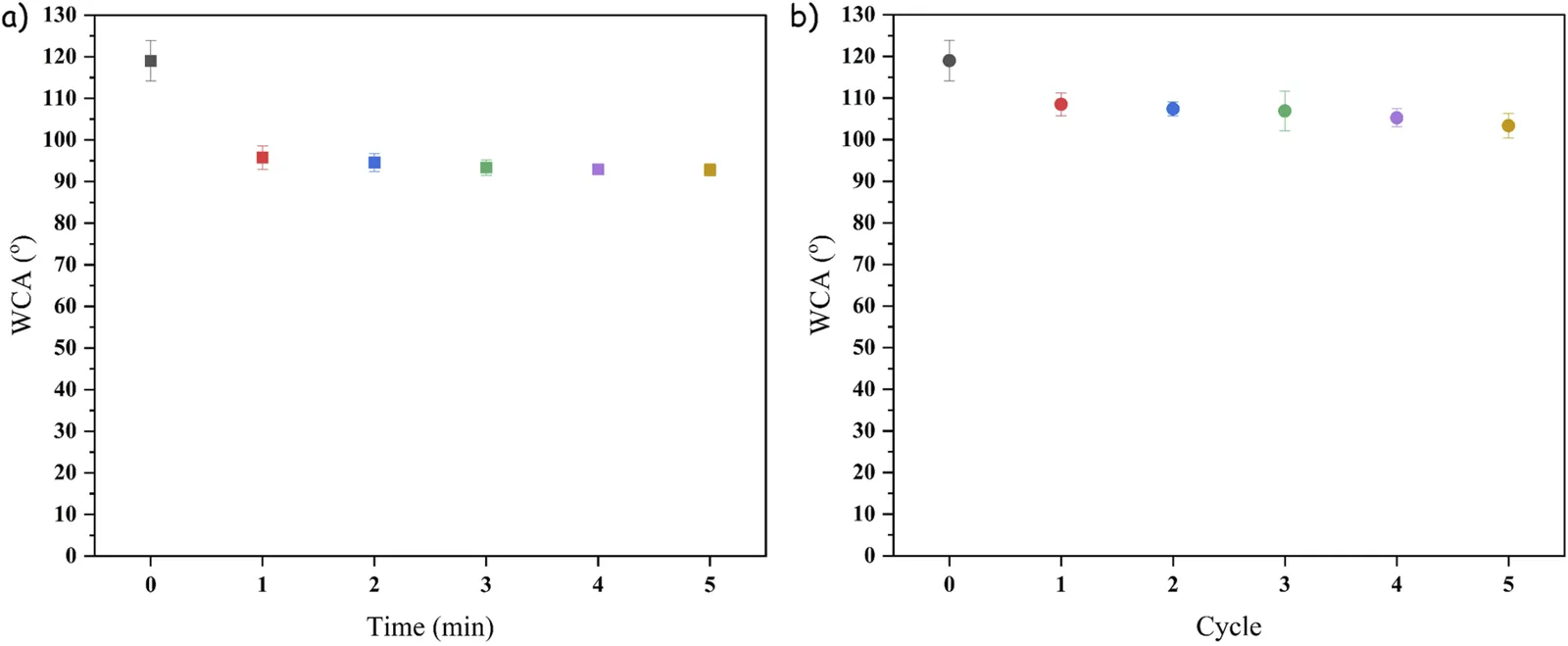
Stability tests of final PDA-CQD-PDMS coatings (a) ultrasonic bath test (b) water impact test.

To further evaluate the durability of the coatings under harsh chemical conditions, the optimized coatings were exposed to solutions at pH 2 and 12. For this purpose, the prepared coatings were immersed in ammonia and HCl solutions for different periods, and the water contact angle was measured as an indicator of changes in surface wettability and coating stability. The results of the alkaline and acidic stability tests are presented in Fig. 10a and b, respectively. As shown in Fig. 10a, the coatings exhibited relatively higher stability under alkaline conditions. After 120 min of exposure to the ammonia solution, the contact angle decreased from the initial value of 119° to approximately 103°, indicating that the hydrophobic character of the coating was largely retained. In contrast, the coatings showed lower stability under acidic conditions (Fig. 10b), with the contact angle decreasing to approximately 92° after 120 min of exposure. These results indicate that the prepared coatings exhibit better chemical stability under strongly alkaline conditions than under acidic conditions.

**Fig. 10:**
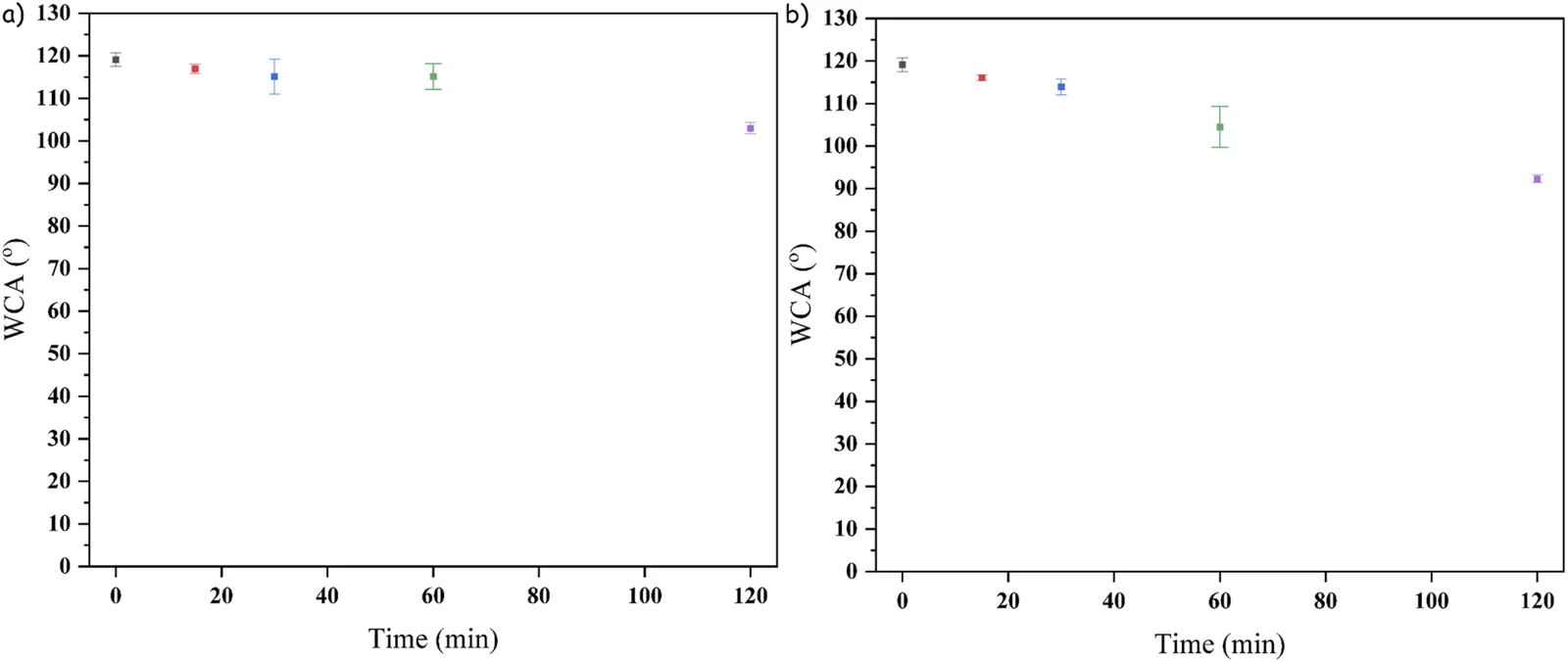
Chemical stability tests of final PDA-CQD-PDMS coatings (a) alkaline conditions (b) acidic conditions.

## 4. Conclusions

In this study, the effects of four nanoparticle types with distinct morphologies, namely CQDs, SiO_2_, HNTs, and GO on dopamine polymerization and the resulting properties of PDA coatings were investigated. The liquid-phase analyses confirmed that nanoparticle incorporation can significantly influence dopamine polymerization. FESEM observations further demonstrated that nanoparticle type and morphology directly affect the size and distribution of PDA-derived particles. Image-based particle size analysis showed that the largest particles were obtained for the PDA-CQD system, with an average diameter of 323 ± 65 nm. Despite the enhanced polymerization in liquid phase observed in the presence of SiO_2_, HNT, and GO compared with pure dopamine and CQDs, the final coatings produced from these systems were generally thinner. This behavior was attributed to the preferential polymerization and deposition of PDA on nanoparticle surfaces rather than on the substrate, which reduces the fraction of strongly adhered material remaining after rinsing. Among the investigated formulations, the PDA-CQD coating prepared at 2 mg mL^−1^ was selected as the optimal sample. This selection was based on the combined outcomes of water contact angle measurements, optical darkening (used as a thickness-related indicator), and AFM roughness analysis. Subsequently, a PDMS top layer was applied to this optimized coating, yielding a hydrophobic surface with a water contact angle of approximately 119°. Mechanical durability assessments, including ultrasonication and repeated water impact, showed that the coatings retained hydrophobicity despite a moderate decrease in contact angle, indicating satisfactory resistance to mechanical loading. Furthermore, exposing the optimized coating to harsh chemical conditions showed moderate chemical resistance. Overall, these findings demonstrate that incorporating nanoparticles with suitable morphology, particularly CQDs, not only modulates dopamine polymerization but also enables the fabrication of coatings with tunable wettability, ranging from hydrophilic to hydrophobic. Given the biocompatible nature of PDA, such coatings may warrant further investigation for biomedical applications including drug-delivery platforms, anti-adhesion (cell/bacterial) surfaces, and engineered interfaces requiring controlled wettability. In addition, the achieved combination of surface roughness and hydrophobicity suggests potential for industrial uses such as self-cleaning and anticorrosion coatings. With further research and development, the prepared coatings could be further optimized for superhydrophobic and self-cleaning applications, as well as for biomedical applications where hydrophobic surfaces are required.

## Conflicts of interest

The authors declare that they have no known competing financial interests or personal relationships that could have appeared to influence the work reported in this paper.

## Supplementary Material

RA-OLF-D6RA07246A-s001

## Data Availability

Data will be made available upon request. Supplementary information (SI) is available. See DOI: https://doi.org/10.1039/d6ra07246a.
