# Supplementary material for: Nanoparticle type-dependent regulation of polydopamine coating formation and wettability
Source: RSC Adv. 2026 Oct 7;16(46):60597–610. doi: 10.1039/d6ra07246a (PMC13643528; doi:10.1039/d6ra07246a)
Supplement: Supplementary file 1 [file RA-016-D6RA07246A-s001.pdf]

Supplementary Information (SI) for RSC Advances.
This journal is © The Royal Society of Chemistry

Supplementary Information

# Nanoparticle Type-Dependent Regulation of Polydopamine Coating Formation and Wettability

Meysam Saberi[1,+], Ebrahim Jalilnia[1,+], Rozita Kazemi[1], Amir Rezvani-Moghaddam[1,*], Hossein Yazdani-Ahmadabadi[2], Mehdi Salami-Kalajahi[1]

[1] *Faculty of Polymer Engineering, Sahand University of Technology, P.O. Box: 51335-1996, Tabriz, Iran*

[2] *Department of Chemistry, University of British Columbia, P.O, Box: V6T 1Z3, Vancouver, British Columbia, Canada*

[+] *Meysam Saberi and Ebrahim Jalilnia contributed equally to this work and share first authorship.*

Corresponding Author E-Mail: arezvani@sut.ac.ir

To employ CQDs in this study, they were synthesized following the procedure previously reported by the INKOATLAB research group [1]. A key feature of CQDs is their photoluminescent behavior: they absorb UV radiation and, due to their electronic structure, emit in the visible region. To verify successful synthesis, the filtered and diluted CQD dispersion was examined under excitation at 240, 250, 260, and 270 nm. As shown in Fig. S1, all excitation wavelengths produced a distinct emission maximum at ∼450 nm, confirming successful CQD formation. Since the synthesis protocol was identical to that previously reported by our research group [1], the synthesized CQDs were expected to exhibit a comparable average particle size of approximately 5 nm.

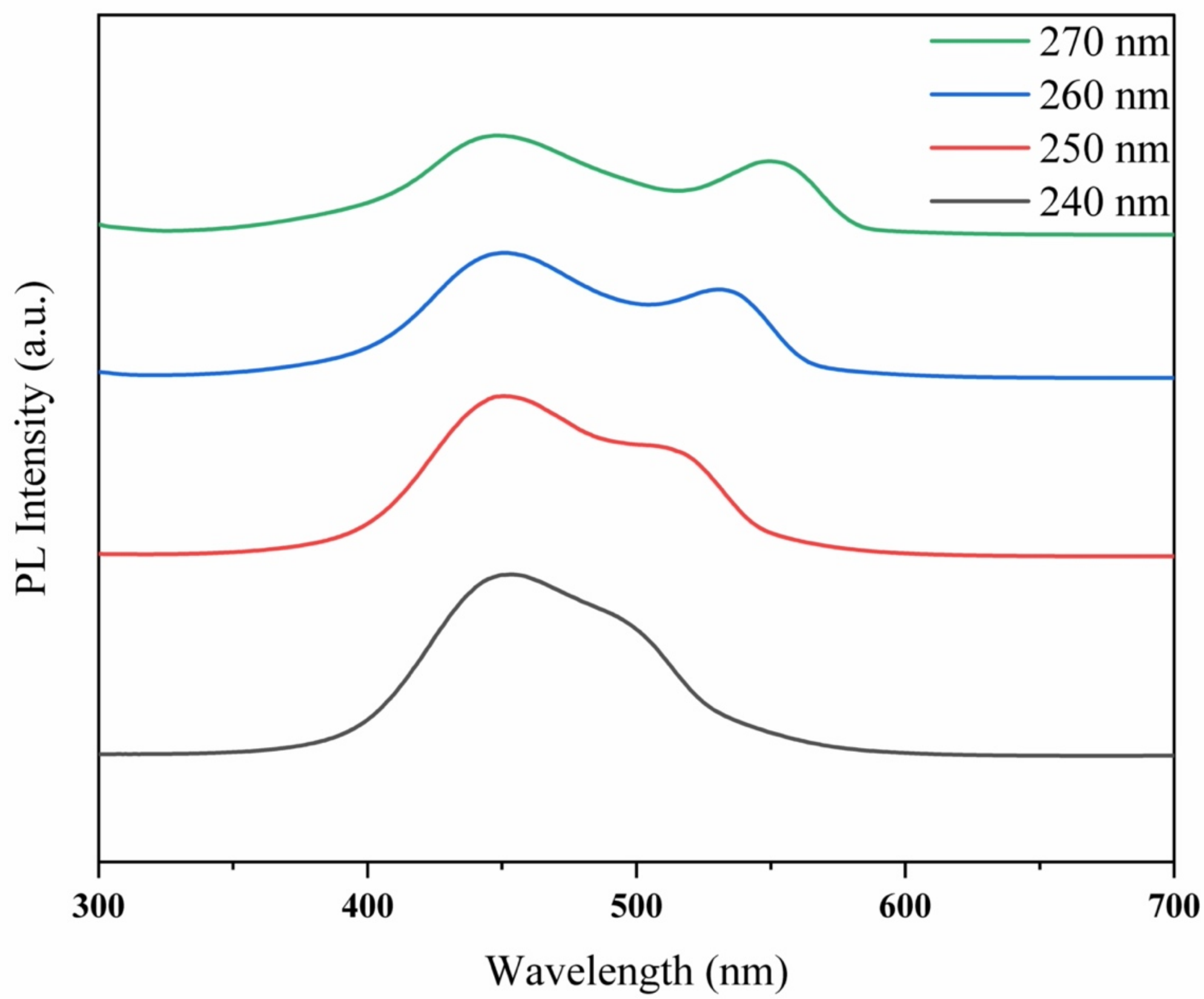


*Figure S1 PL results of the synthesized CQDs under excitation wavelengths of 240, 250, 260, and 270 nm.*

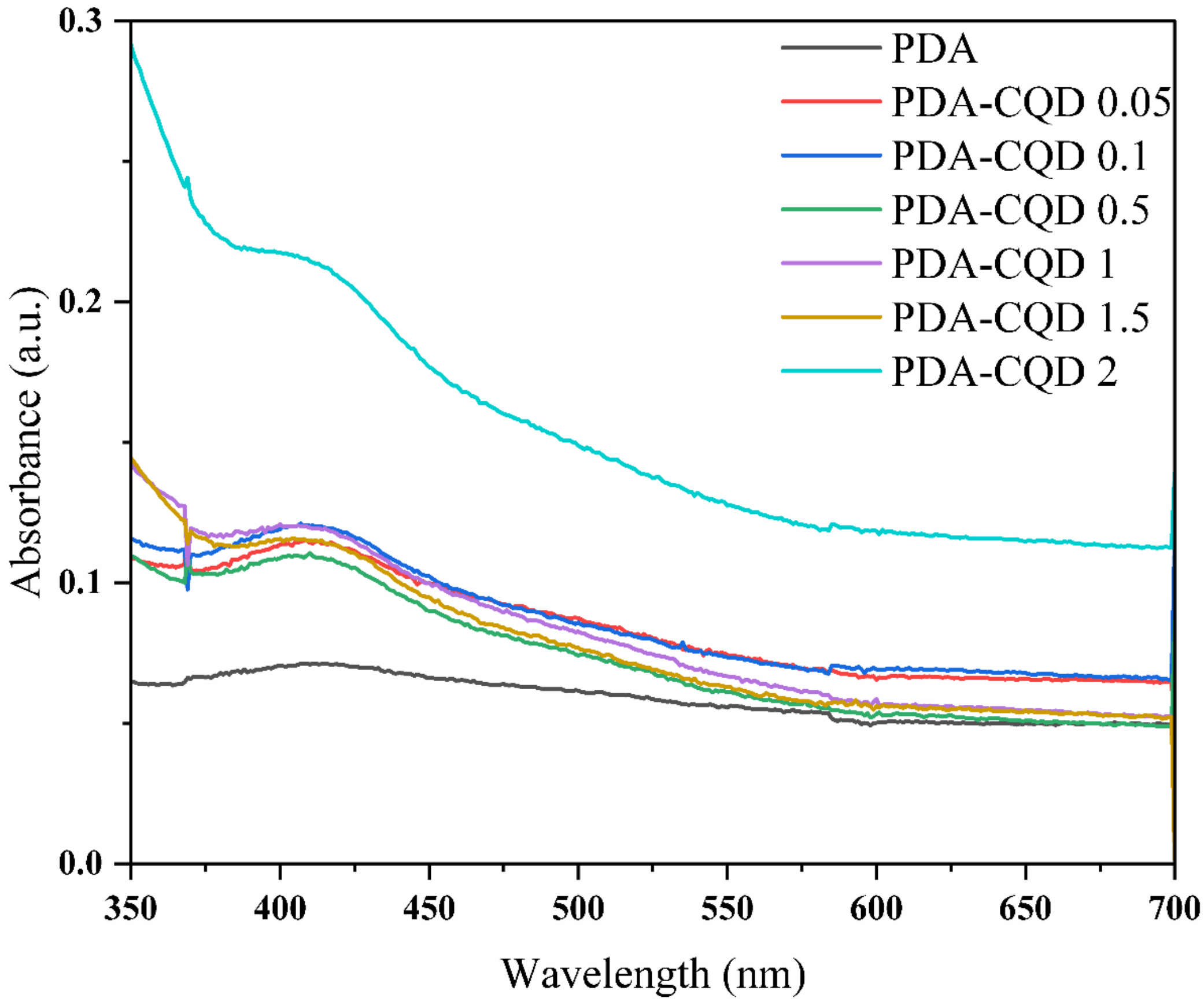


*Figure S2: UV-Vis spectra of PDA samples containing CQD (0.05 – 2 mg.ml$^{-1}$)*

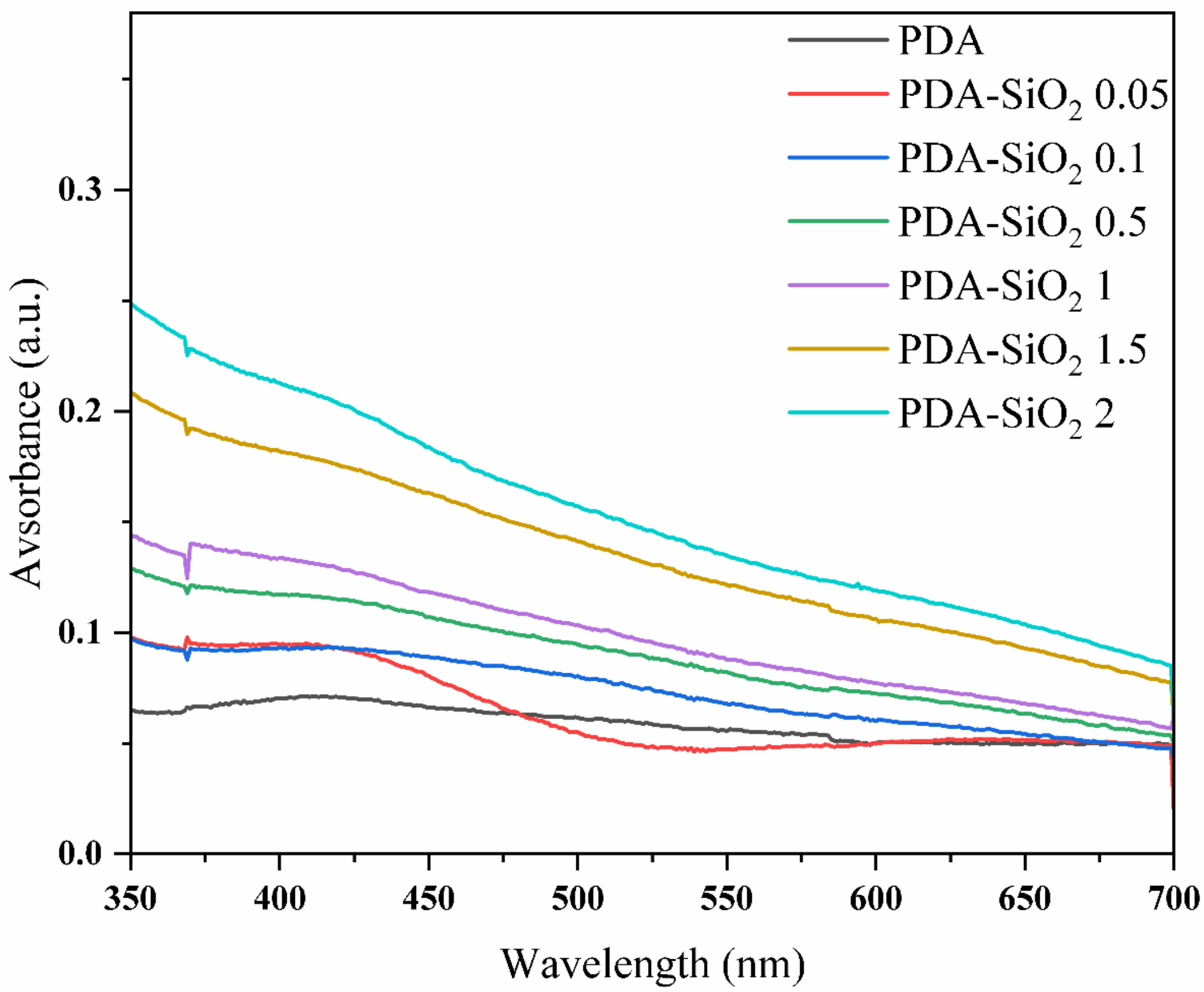


*Figure S3: UV-Vis spectra of PDA samples containing $SiO_2$ (0.05 – 2 $mg.ml^{-1}$)*

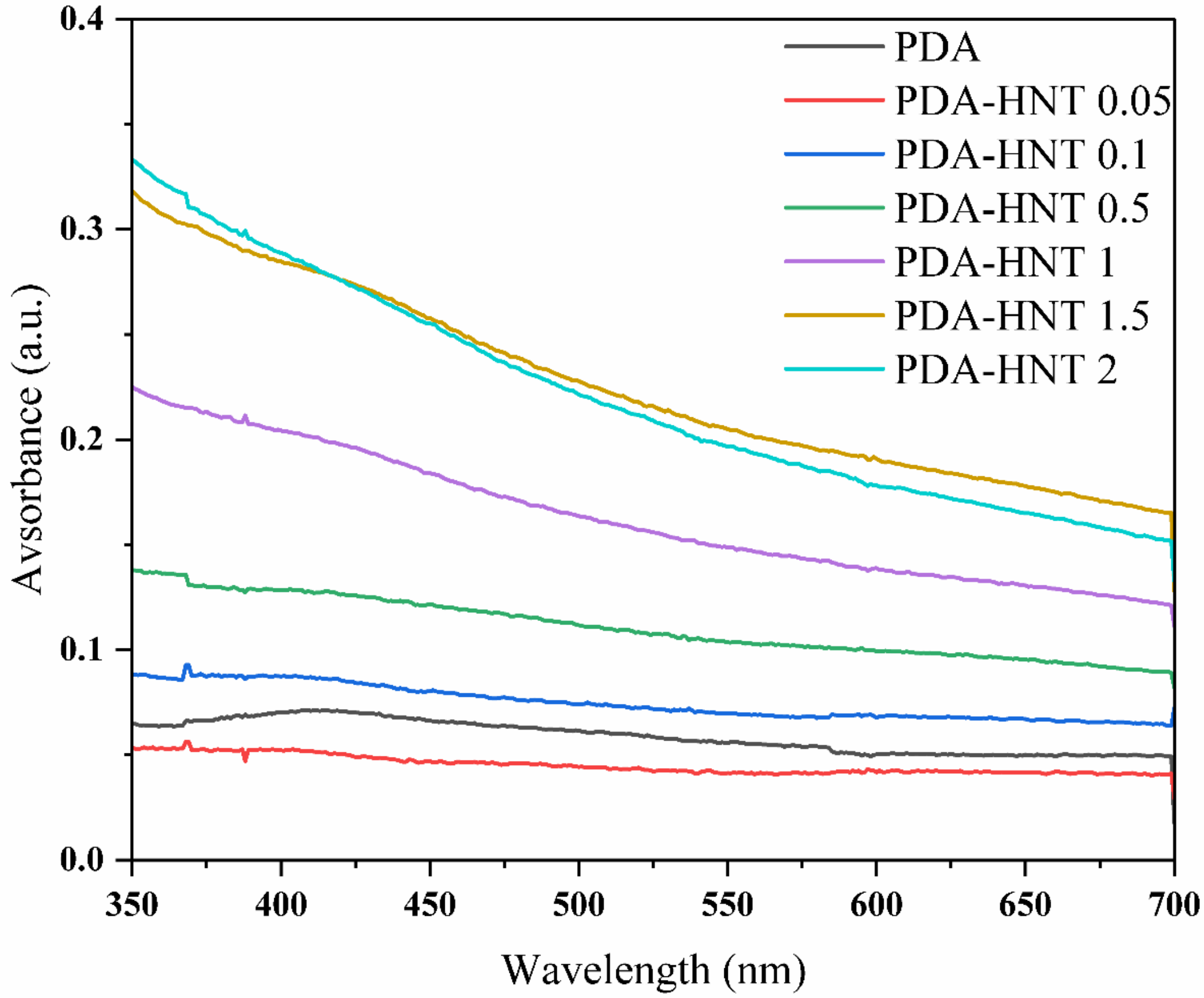


*Figure S4: UV-Vis spectra of PDA samples containing HNT (0.05 – 2 $mg.ml^{-1}$)*

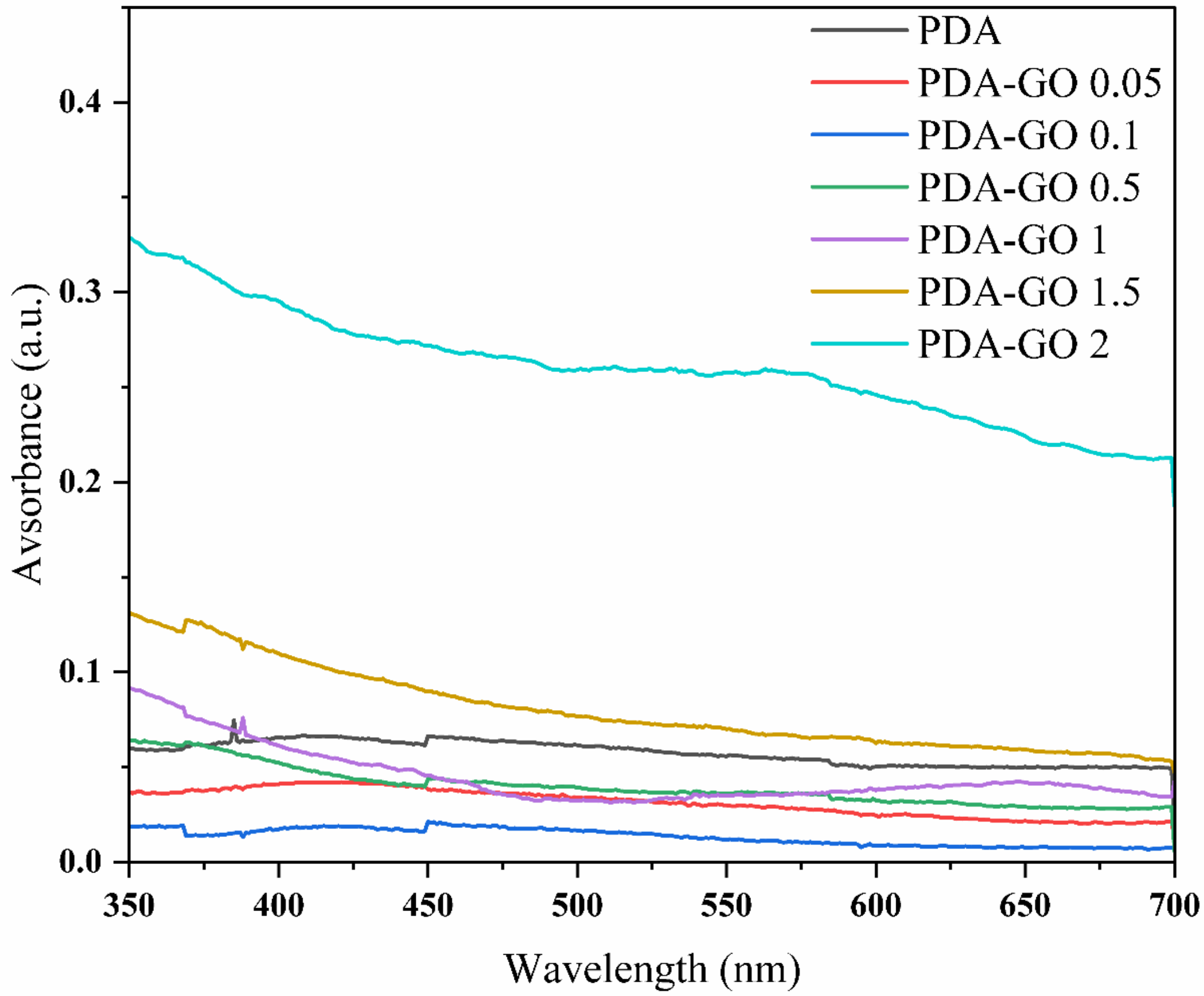


*Figure S5: UV-Vis spectra of PDA samples containing GO (0.05 – 2 mg.ml$^{-1}$)*

The FTIR spectra of the dried products obtained from dopamine polymerization in the presence of the different nanoparticles are presented in Figure S6. The spectra exhibit characteristic bands associated with functional groups originating from dopamine/PDA, together with bands attributable to the respective nanoparticles. The broad absorption bands observed in the 3300–3600 cm$^{-1}$ region are assigned to O–H/N–H stretching vibrations. The bands in the 2850–2950 and 3000–3100 cm$^{-1}$ regions are associated with the stretching vibrations of aliphatic and allylic C–H groups, respectively. The characteristic bands observed at approximately 1506 and 1660 cm$^{-1}$ are attributed to C=C-related vibrations of aromatic structures. In addition, the bands in the 700–800 cm$^{-1}$ region are associated with

vibrations of reduced aromatic structures . For the silica- and halloysite-containing samples, characteristic Si–O–Si vibrations are also evident. The FTIR spectrum of the graphene oxide-containing sample additionally shows a characteristic carbonyl (C=O) band at approximately 1703 cm$^{-1}$.

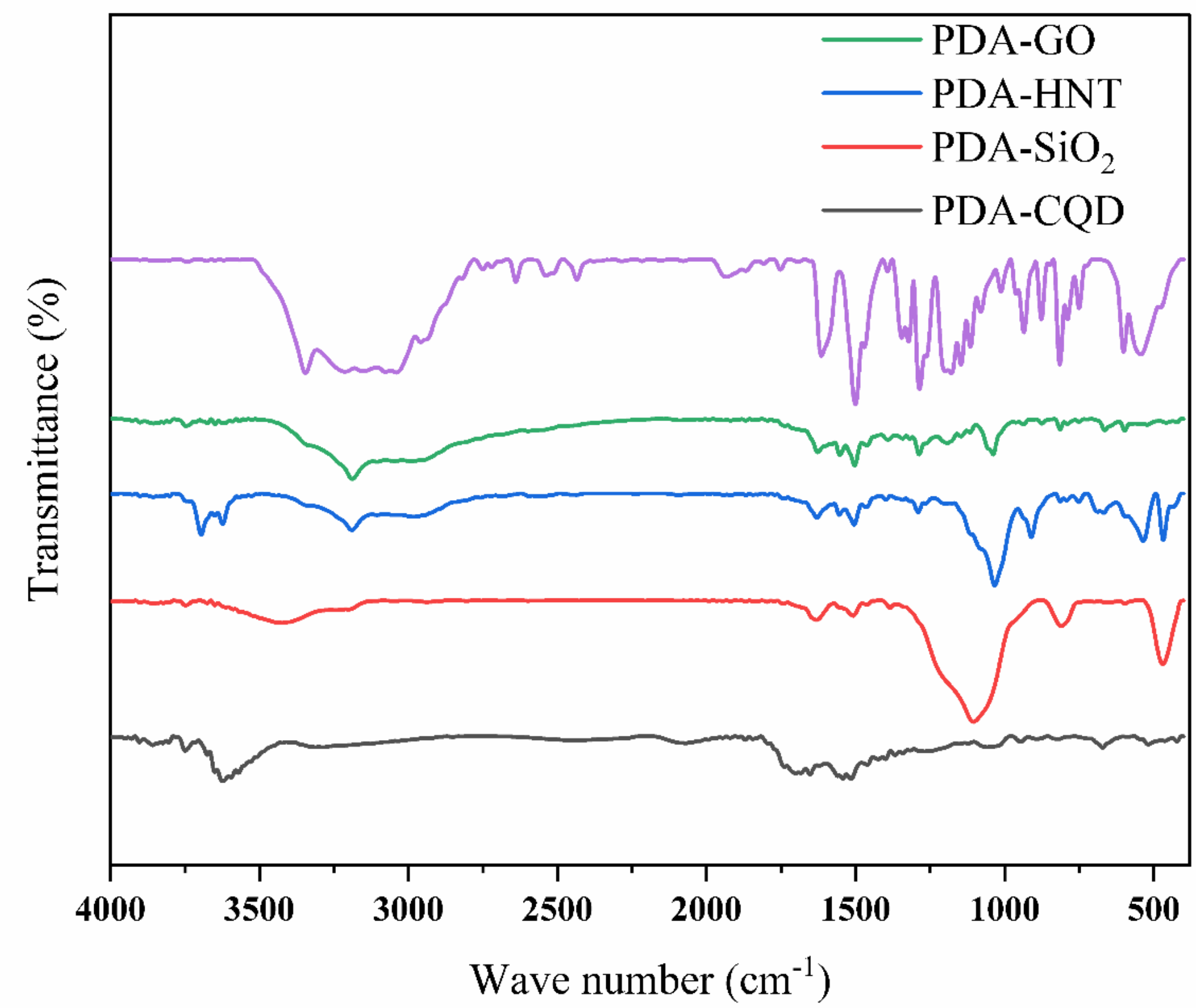


*Figure S6 FTIR spectra of dried PDA-NP powders after polymerization*

To further examine the formation of PDA-containing coatings, ATR-FTIR measurements were performed directly on the coating surfaces prepared at nanoparticles' concentrations of 0.5 and 2 mg mL$^{-1}$. The corresponding spectra are presented in Figure S7. A distinct band in the 700–800 cm$^{-1}$ region, also observed in the dried polymerization products, is associated with vibrations of reduced aromatic structures in PDA. Although the other characteristic PDA bands are less pronounced in the ATR spectra, their overall spectral features are consistent with previously reported FTIR characteristics of PDA and support the formation of PDA coatings in the presence of the investigated nanoparticles [2].

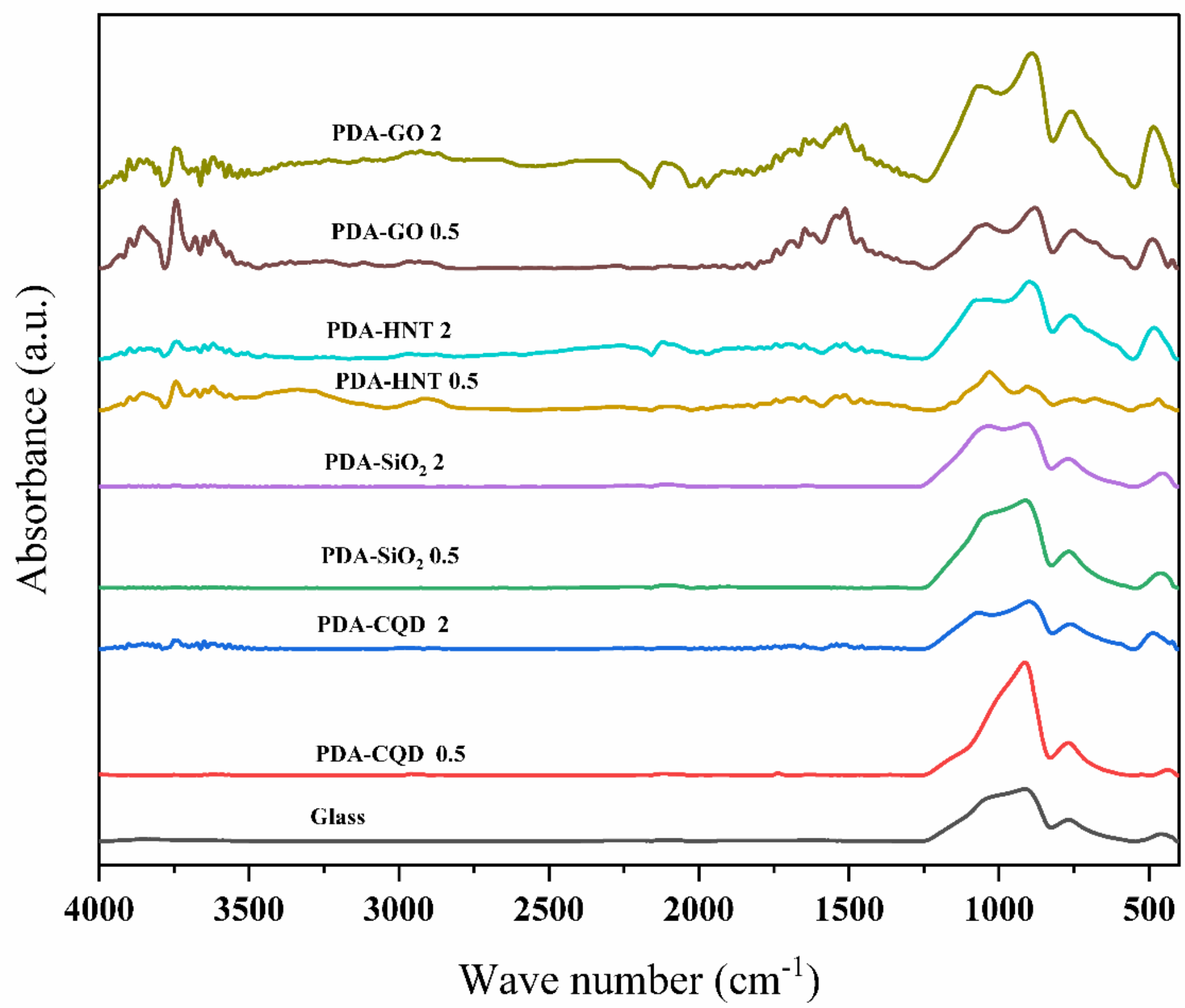


Figure S7. FTIR-ATR spectra of the coating prepared at nanoparticles' concentrations of 0.5 and 2 mg.mL$^{-1}$

## References


[1] Mirlou-Miavagh, F., A. Rezvani-Moghaddam, H. Roghani-Mamaqani, and U. Sundararaj, *Comparative study of synthesis of carbon quantum dots via different routes: Evaluating doping agents for enhanced photoluminescence emission.* Progress in Organic Coatings, 2024. **191**: p. 108445.

[2] Khosravi, H., R. Naderi, and B. Ramezanzadeh, *Synthesis and application of molybdate-doped mussel-inspired polydopamine (MI-PDA) biopolymer as an effective sustainable anti-corrosion substance for mild steel in NaCl solution.* Biomass Conversion and Biorefinery, 2024. **14**(21): p. 27557-27573.
